# Clinical and economic impact of genome-wide non-invasive prenatal testing (NIPT) as a first-tier screening method compared to targeted NIPT and first-trimester combined testing: A modeling study

**DOI:** 10.1371/journal.pmed.1004790

**Published:** 2025-11-05

**Authors:** Lisanne van Prooyen Schuurman, Harry J. de Koning, Eva Meier, Robert-Jan H. Galjaard, Nicolien T. van Ravesteyn

**Affiliations:** 1 Department of Public Health, Erasmus MC, University Medical Center Rotterdam, Rotterdam, the Netherlands; 2 Department of Clinical Genetics, Erasmus MC, University Medical Center Rotterdam, Rotterdam, the Netherlands; 3 Department of Ethics, Law & Humanities, Amsterdam UMC Location Vrije Universiteit, Amsterdam, the Netherlands; Stanford University, UNITED STATES OF AMERICA

## Abstract

**Background:**

Evidence on the diagnostic yield of genome-wide non-invasive prenatal testing (GW-NIPT) is growing, but its comparative clinical and economic impact as a first-tier screening strategy for fetal chromosomal abnormalities remains unassessed. We compared GW-NIPT with targeted NIPT and first-trimester combined testing (FCT), in a Dutch setting where all pregnancies also undergo a routine second-trimester anomaly ultrasound scan (scan), to guide policymakers on optimal prenatal screening approaches.

**Methods and findings:**

We developed a decision-analytic model for a cohort of 175,000 pregnancies, reflecting the Dutch obstetric population. All strategies screened for common trisomies 21 (Down syndrome), 18 (Edwards syndrome), and 13 (Patau syndrome); GW-NIPT additionally considered rare autosomal trisomies and structural aberrations. Model inputs were based on the TRIDENT-2 study data and historical FCT data. Base-case unit costs were €166 (scan), €191 (FCT), and €350 (NIPT). Sensitivity analyses were conducted to account for uncertainties in model parameters and potential country-specific variations. Outcomes included total screening costs, number of fetal chromosomal abnormalities diagnosed, number of invasive procedures, and expected procedure-related euploid fetal losses. We summarized economic results as cost per diagnosed case and incremental cost per additional diagnosis across strategies. GW-NIPT yielded the highest number of diagnoses (545) versus targeted NIPT (514) and FCT (452), and the lowest cost per diagnosed case (€152,785), compared with targeted NIPT (€159,852) and FCT (€170,050). Invasive tests required per diagnosis were lower for GW-NIPT and targeted NIPT (both 6) than for FCT (13), implying a lower risk of procedure-related miscarriage (iatrogenic miscarriage). Sensitivity analyses indicated that test uptake and unit costs strongly influenced outcomes. GW-NIPT remained the most favorable in terms of cost per diagnosis for NIPT prices up to €467. Key limitations include the use of a decision-analytic model without quality-of-life outcomes and the lack of comparisons against explicit cost-effectiveness thresholds. Therefore, the results should be interpreted as relative clinical and economic comparisons rather than cost-effectiveness judgements.

**Conclusions:**

Among the strategies evaluated, first-tier GW-NIPT had the greatest diagnostic yield and the lowest cost per diagnosis, improving detection rates and supporting reproductive autonomy at lower costs. Implementation decisions should also consider local pricing, laboratory capacity, and counseling resources. Future research that links screening outcomes to long-term health consequences (e.g., quality-adjusted life years or life-years), healthcare utilization, costs, and psychosocial outcomes will enable formal cost-effectiveness evaluations and support further refinement of prenatal screening policy.

## Author summary

**Why was this study done?:**

- Prenatal screening aims to detect fetal chromosomal abnormalities, such as Down syndrome, early in pregnancy to help expectant parents make informed reproductive choices.
- Until recently, most countries used established tests such as the first-trimester combined test. The introduction of non-invasive prenatal testing (NIPT), a simple blood test, has changed the field. NIPT can be used in a targeted way to screen for common syndromes (Down, Edwards, and Patau) or in a genome-wide format (GW-NIPT), which can also identify other serious chromosomal abnormalities.
- While GW-NIPT has the potential to detect a broader range of genetic conditions, its clinical and economic impact as a first-line test are not yet well understood.
- This study evaluated how offering GW-NIPT to all pregnant women could affect detection rates and healthcare costs.

**What did the researchers do and find?:**

- The authors used a simulation model based on data from 175,000 pregnancies in the Dutch national screening program to compare various prenatal screening strategies.
- The results showed that offering GW-NIPT detects 20% more cases of chromosomal abnormalities than first-trimester combined testing and reduces the number of invasive procedures by 45%, lowering the risk of procedure-related miscarriage.
- Although the GW-NIPT is more expensive, it detects more cases and leads to fewer unnecessary positive results that would require follow-up invasive testing, resulting in a lower cost per diagnosis.

**What do these findings mean?:**

- Implementing GW-NIPT as a routine first-tier screening test in prenatal care can improve early detection of fetal conditions while minimizing risks to pregnant women.
- While introducing GW-NIPT may increase overall costs, it offers greater efficiency and has a lower cost per case detected. This supports reproductive autonomy by offering expecting parents better information and may inform prenatal screening policies.
- A limitation is that the impact on long-term healthcare costs or the quality of life of affected individuals and families is still largely unknown, and therefore, a formal cost-effectiveness analysis with outcomes comparable across other health interventions was not feasible.

## Abstract 3

In this modelling study, Lisanne van Prooyen Schuurman and colleagues compared the clinical and economic impact of different prenatal testing methods.

## Introduction

Chromosomal abnormalities affect about 1 in 150 pregnancies, leading to severe phenotypic abnormalities in the child, such as developmental delays and congenital anomalies [1]. Prenatal screening allows expectant parents to test for these abnormalities, facilitating informed reproductive decisions [2]. Across Europe, many countries offer first-trimester screening for common trisomies: Down syndrome (trisomy 21; T21), Edwards syndrome (trisomy 18; T18), and Patau syndrome (trisomy 13; T13), primarily using the first-trimester combined test (FCT) [3,4]. While FCT has a reasonable sensitivity (0.75–0.95), it has a relatively high false-positive rate that increases with maternal age (0.02–0.084) [5,6].

In recent years, prenatal screening has been revolutionized by the introduction of non-invasive prenatal testing (NIPT). NIPT analyses cell-free DNA (cfDNA) in maternal plasma [7], and can be conducted either targeted, focusing on common trisomies, or as genome-wide NIPT (GW-NIPT), which detects additional chromosomal abnormalities (additional findings) [8,9]. These additional findings can be categorized into rare autosomal trisomies (RATs), structural aberrations (SAs), and complex profiles [8]. RATs are chromosomal abnormalities where there are three copies of one of the non-sex (autosomal) chromosomes instead of the usual two. Unlike the common trisomies, RATs involve other, less frequently affected chromosomes. RATs are often associated with developmental problems, such as fetal growth restriction, congenital anomalies, pregnancy complications, and, in some cases, neurodevelopmental impairment, but the effects can vary widely depending on the specific chromosome involved [8,9]. SAs are chromosomal abnormalities that involve changes in the structure of one or more chromosomes, including deletions (loss of part of a chromosome segment), and duplications (gain of a part of a chromosome segment). SAs can disrupt normal gene function and lead to genetic disorders and developmental problems. In the context of prenatal or genetic testing, a “complex profile” refers to a sample with chromosomal aberrations consisting of multiple losses and gains of whole, or parts, of chromosomes. These complex profiles often originate from maternal (acquired) aberrations [10,11].

While GW-NIPT enhances reproductive choices by detecting additional severe fetal abnormalities, it also detects placental and maternal aberrations due to the nature of the tested material [8–10], which some perceive as drawbacks compared to targeted NIPT. Despite growing evidence of GW-NIPT’s clinical benefits, uncertainties about its economic implications remain a barrier to broader adoption [12].

NIPT is relatively expensive, estimated at €350 (380 USD) compared to €191 (208 USD) for FCT in the Dutch healthcare context during the TRIDENT-2 (TRIal by Dutch laboratories for Evaluation of Non-invasive prenatal Testing) study (2017–2023). For NIPT performed in a clinical context other than routine prenatal screening, reimbursement values decreased from €915 to €653 (995–710 USD) between 2021 and 2024. The cost difference between NIPT and FCT has raised concerns within the field of prenatal screening about the potential strain on budgets if NIPT becomes the preferred method.

Previous evaluations of costs and effects have primarily focused on targeted NIPT rather than GW-NIPT [13,14], or expanded NIPT for specific abnormalities such as sex chromosome aneuploidies and microdeletion syndromes [15–17]. Moreover, these analyses have not included the second-trimester ultrasound, which is a routine part of prenatal care in almost all countries [18,19] and can independently detect structural anomalies often linked to genetic conditions. Excluding the ultrasound scan risks overestimating the added value of genetic screening tests. The current study addresses this gap by assessing and comparing the clinical and economic impact of GW-NIPT, targeted NIPT, and FCT, alongside second-trimester ultrasound screening in the Dutch healthcare context.

It is important to clarify that this analysis is not a conventional cost-effectiveness evaluation. Standard health outcome measures such as quality-adjusted life years (QALYs) are not used. Assigning QALYs in prenatal screening is challenging due to the complex ethical and methodological issues involved. Prenatal screening primarily provides information rather than direct health benefits, outcomes include pregnancy termination or preparation for affected offspring, and it is difficult to determine whose QALYs to consider (child, parents, or society). Furthermore, the heterogeneity of genetic conditions complicates assigning meaningful and nondiscriminatory QALY values, but also long-term costs are often uncertain or unknown. We therefore report cost per case detected (diagnosis) and incremental cost per additional diagnosis. Although this limits comparison with cost-effectiveness analyses in other healthcare domains, it aligns with standard practice in prenatal screening evaluations and captures the primary objective of maximizing detection of serious fetal conditions at the lowest cost [20]. Because no recognized threshold exists for interpreting cost per anomaly detected, we present results as relative clinical and economic comparisons across strategies rather than absolute judgments of cost-effectiveness.

The modeled base-case scenario follows the Dutch TRIDENT-2 protocol, offering women a choice between genome-wide and targeted NIPT testing [21,22]. This approach reflects real-world implementation [23], and is grounded in empirical data from the Dutch screening program, enhancing the accuracy and relevance of the model.

Our primary objective is to compare the clinical yield and total screening program costs of GW-NIPT, targeted NIPT, and FCT, each alongside routine second-trimester ultrasound, in the Dutch setting, reporting cost per diagnosis and incremental cost per additional diagnosis. The findings from this study can inform policymakers about the clinical and economic implications of adopting GW-NIPT as a primary prenatal screening strategy and guide future decisions regarding program design and reimbursement policy.

## Methods

### Ethics and consent

This study used aggregated data from the Dutch TRIDENT-2 national screening programme and published sources and involved no interaction or intervention with human participants; therefore, additional ethics committee review for this modeling analysis was not required. TRIDENT-2 obtained informed consent from participating women as part of the national programme.

This study is reported as per the Strengthening the Consolidated Health Economic Evaluation Reporting Standards 2022 (CHEERS 2022) Statement (S1 Checklist).

### Screening strategies

This study assessed the clinical and economic impact of prenatal screening for chromosomal abnormalities using GW-NIPT, compared with targeted NIPT and FCT, within the Dutch obstetric population. Each strategy incorporated the offer of a genetic screening test (NIPT or FCT) alongside a second-trimester anomaly scan, which can detect chromosomal anomalies through structural fetal anomalies. A strategy with only the second-trimester anomaly scan was also included to highlight the added value of genetic screening beyond ultrasound. In the GW-NIPT strategy, pregnant women could choose between targeted and genome-wide analyses. GW-NIPT, as implemented in the Dutch TRIDENT studies, is designed to detect common aneuploidies (T21, T18, T13) as well as RATs and SAs ≥ 7 megabases (Mb), referred to here as additional findings. Sex chromosome aneuploidies were not analyzed, consistent with the TRIDENT protocol. Twin and multiple pregnancies were excluded due to insufficient data for robust modeling.

### The model

**Fig 1:**
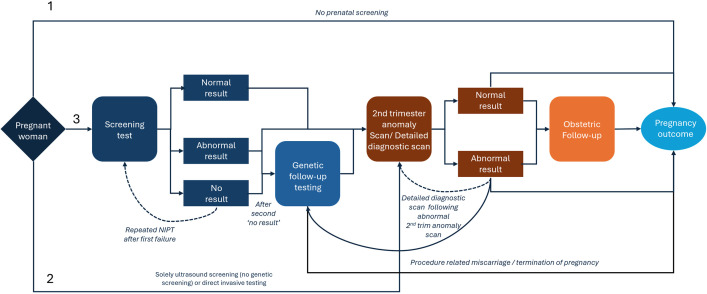
Schematic representation illustrating the potential screening pathways within the decision-analytic model. The specific screening test and associated probabilities in the model are contingent upon the evaluated screening strategy, as detailed in Table 1. Women who opt for invasive testing without prior screening are presumed to undergo a detailed diagnostic scan before the invasive procedure. Genetic follow-up testing may involve invasive testing and/or genetic testing in the mother, depending on the identified chromosomal aberrations from the screening test. Following an abnormal result in the second-trimester anomaly scan, a detailed diagnostic scan is conducted to confirm or rule out the findings. Obstetric follow-up includes three scans for fetal biometry measurement and placental functioning between 26 and 36 weeks of gestation.

The flow diagram in Fig 1 outlines possible test participation routes and outcomes, independent of the screening strategy. These pathways were developed with input from relevant specialists, including clinical geneticists, lab specialists, and gynecologists. Four main routes for pregnant women and their partners were considered: no prenatal screening, direct invasive testing, second-trimester anomaly scan only, and genetic prenatal screening (GW-NIPT, targeted NIPT, or FCT) in addition to the anomaly scan. All screening methods aimed to detect T21, T18, and T13, with GW-NIPT also targeting additional findings. A small percentage opted for direct invasive testing due to known elevated risk. In the event of a “no result” on the initial NIPT (often due to low fetal fraction or cfDNA variability), a repeat NIPT was offered. If the second attempt again yielded no result, women were referred for invasive testing [24].

**Table 1:** Model inputs.

Variable	Base case value	PSA range	DSA range	Ref
Population				
Singleton pregnancies at 12 weeks GA	175,000	–	–	[27]
Age distribution		–	–	[27]
Prevalence of common trisomies in first trimester	Age-specific, see S1 Table	–	–	[26]
Prevalence RATs in first trimester	0.0018	±10%^a^	±10%^a^	[8]
Proportion of RATs confirmed in fetus	0.08	–	–	[8]
Proportion of RATs confirmed in placenta	0.91	–	–	[8]
Proportion of RATs confirmed in mother	0.01	–	–	[8]
Prevalence SAs in first trimester	0.0017	±10%^a^	±10%^a^	[8]
Proportion of SAs confirmed in fetus	0.44	–	–	[8]
Proportion of SAs confirmed in placenta	0.06	–	–	[8]
Proportion of SAs confirmed in mother	0.5	–	–	[8]
Pretest counseling scope	0.902	0.865–0.928	–	[30]
Test uptake				
Uptake invasive testing without prior screening	0.0043	0.0036–0.0050	±20%^a^	[31]
Uptake screening tests	Age-specific, see S2 Table	±10%	±20%/ 0.1–1.0^a^^,^^b^	[29]
Uptake second-trimester anomaly scan	0.847	0.821–0.866	±20%^a^	[30]
Uptake detailed diagnostic scan following a positive screening result	1	–	–	Assumption
Uptake invasive testing following a positive NIPT result for T21, T18, or T13	0.924	±10%^a^	±20%^a^	[32]
Uptake invasive testing following a positive FCT	0.88	0.84–0.92	±20%^a^	[31]
Uptake genetic follow-up testing following a positive screen result for a RAT	0.75 invasive testing; 0.42 genetic testing in women	±10%^a^	±20%^a^	[8]
Uptake genetic follow-up testing following a positive screen result for a SA	0.68 invasive testing; 0.83 genetic testing in women	±10%^a^	±20%^a^	[8]
Uptake invasive testing in case of ultrasound anomalies	0.66	0.62–0.70	±20%^a^	[31]
Test characteristics				
Sensitivity FCT T21	0.84	0.76–0.95	0.76–0.95	[33]
Sensitivity FCT T18	0.92	0.85–0.95	0.85–0.95	[33]
Sensitivity FCT T13	0.84	0.75–0.90	0.75–0.90	[33]
False-positive FCT	0.055	0.02–0.084	0.02–0.084	[33]
Failure rate first NIPT draw	0.015	0.014–0.02	0.014–0.02	[9,32]
Failure rate second NIPT draw	0.14	±10%^a^	±10%^a^	[32]
Sensitivity NIPT T21	0.988	0.9781–0.9934	0.9781–0.9934	[34]
Sensitivity NIPT T18	0.9883	0.9545–0.9997	0.9545–0.9997	[34]
Sensitivity NIPT T13	1.0	0.999–1.00	0.999–1.00	[34]
Sensitivity NIPT RAT and SA	1.0	–	–	Assumption
False-positive NIPT	0.0004	0.0002–0.0008	0.0002–0.0008	[34]
Sensitivity 2^nd^ trimester anomaly scan T21^c^	0.43	0.40–0.69	0.40–0.69	[35–37]
Sensitivity 2^nd^ trimester anomaly scan T18^c^	0.93	±10%^a^	±10%^a^	[38]
Sensitivity 2^nd^ trimester anomaly scan T13^c^	0.95	±10%^a^	±10%^a^	[39]
Sensitivity 2^nd^ trimester anomaly scan fetal RAT^c^	0.15	±10%^a^	±10%^a^	[8]
Sensitivity 2^nd^ trimester anomaly scan fetal SA^c^	0.50	±10%^a^	±10%^a^	[8]
Screen-positive 2^nd^ trimester anomaly scan (non-genetic)^c^	0.044	±10%^a^	±10%^a^	[30]
Proportion of anomalies confirmed by detailed diagnostic scan (genetic)	1.00	±10%^a^	±10%^a^	Assumption
Proportion of anomalies confirmed by detailed diagnostic scan (non-genetic)	0.38	±10%^a^	±10%^a^	[30]
Sensitivity and specificity invasive test	1	–	–	Assumption
Birth outcomes				
Risk of iatrogenic miscarriage	0.0012	–	–	[40]
TOP rate in case of T21	0.88	–	–	[41]
TOP rate in case of T18	0.86	–	–	[42]
TOP rate in case of T13	0.92	–	–	[42]
TOP rate in case of a fetal RAT	0.50	–	–	[8]
TOP rate in case of a fetal SA	0.90	–	–	[8]
TOP rate in case of ultrasound anomalies	0.40	–	–	[43]
General risk of IUFD	0.006	–	–	[44]
Risk of IUFD in case of T21	0.38	–	–	[45]
Risk of IUFD in case of T18	0.70	–	–	[46]
Risk of IUFD in case of T13	0.50	–	–	[46]
Risk of IUFD in case of fetal RAT	0.11	–	–	[8]
Risk of IUFD in case of fetal SA	0.006	–	–	[8]

Abbreviations: FCT, first-trimester combined testing; GA, gestational age; IUFD, intra-uterine fetal demise; NIPT, non-invasive prenatal testing; RATs, rare autosomal trisomies; SAs, structural aberrations; T, trisomy; TOP, termination of pregnancy.

^a^Assumed ranges: either ±10% around the base case value or, for parameters like test uptake that may vary substantially between countries, wide ranges from 0.0 to 1.0.

^b^Screening test uptake was varied around the base case in two ways: (1) ±20% around the age-specific base case values, preserving age-related variation, and (2) uniformly across age groups, ranging from 0.1 to 1.0.

^c^Test characteristics of the detailed diagnostic scan were assumed to be equivalent to those of the second-trimester anomaly scan.

Follow-up strategies depended on the suspected chromosomal abnormality [25]. If structural anomalies were indicated during the second-trimester anomaly scan, women were referred for a detailed diagnostic ultrasound at a tertiary center and, if confirmed, invasive testing was recommended. All abnormal screening results (common trisomy or additional finding) necessitated confirmation through invasive diagnostic tests, which carry the risk of iatrogenic miscarriage (IM). In cases of additional findings, cytogenetic testing on maternal blood was sometimes recommended to rule out maternal origins. Detailed diagnostic scans were advised when relevant for choosing between chorionic villus sampling (CVS) or amniocentesis, clarifying fetal prognosis, or in cases of (suspected) confined placental mosaicism (CPM). Additionally, women with suspected CPM were offered three ultrasound scans between 26 and 36 weeks to monitor fetal growth and placental function. These scans were also conducted after abnormal NIPT results if parents opted against invasive follow-up.

All testing pathways conclude with pregnancy outcomes, including termination of pregnancy (TOP) for confirmed chromosomal abnormalities or ultrasound anomalies, IM, intrauterine fetal demise (IUFD)/stillbirth, or live birth. The risk of IUFD or stillbirth is elevated in pregnancies with chromosomal abnormalities compared to unaffected pregnancies [26].

### Analysis

#### Population.

A theoretical cohort of 175,000 pregnant women, representing the yearly Dutch population at 12 weeks of gestation, was divided into five age groups to account for age-dependent model inputs [27]. The prevalence of common trisomies was derived from literature [26] (S1 Table) and TRIDENT-2 outcomes for RATs and SAs [8] (Table 1). Cases with NIPT results indicating complex profiles are excluded from this study, as they typically involve maternal (acquired) abnormalities [8,10,11]. The model was built and evaluated in R (version 4.2.1) following the Decision Analysis in R for Technologies in Health (DARTH) framework [28]. Model inputs were derived from Statistics the Netherlands, the national prenatal screening database Peridos [29], TRIDENT-2 study data, and existing literature.

#### Test uptake and test characteristics.

Screening uptake reflected FCT and NIPT trends in the Netherlands (S2 Table), with higher NIPT uptake and increasing rates with maternal age. The higher uptake of NIPT (mean: 44%) compared to FCT (mean: 33%) can likely be attributed to its superior test characteristics, which results in a lower likelihood of unnecessary invasive follow-up procedures. Sensitivity and specificity values for screening tests [33,34] and the anomaly scan [35–39] were derived from literature and TRIDENT-2 data [8]. GW-NIPT sensitivity for RATs and SAs was set at 100%, assuming TRIDENT-2 findings mirrored the real prevalence in the general obstetric population, as false-negative rates were not reported. Uptake of the second-trimester anomaly scan after genetic screening is assumed to be 100%, independent of maternal age. Among those not opting for genetic screening, 84.7% choose the anomaly scan [30]. Uptake for a tertiary center detailed diagnostic scan following an abnormal second-trimester anomaly scan was assumed to be 100%. Invasive testing uptake varied with screening results [8,32]. Invasive tests were assumed to have 100% diagnostic accuracy [47]. Women with abnormal FCT results received counseling from a gynecologist and a clinical geneticist if further invasive tests were abnormal. For NIPT, T21, T18, and T13, results were discussed in a basic consultation, while additional findings required a detailed consultation with the clinical geneticist. It was assumed that all women would agree to additional obstetric follow-up if they declined invasive testing or if the fetal or placental origin of the abnormality was confirmed or presumed, and the pregnancy continued.

#### Birth outcomes.

The probability of IM (0.12%) was based on average rates for CVS and amniocentesis [40]. TOP and IUFD probabilities were assumed independent of maternal age but dependent on the specific chromosomal abnormality (Table 1) [8,42–46]. Due to the lack of systematic reporting on birth outcomes for common trisomies in the TRIDENT study, these probabilities were obtained from existing literature.

#### Costs.

Total costs for prenatal screening and diagnosis, from a healthcare perspective, included expenses for screening and diagnostic tests, second-trimester anomaly scans, detailed diagnostic scans, genetic counseling, and additional obstetric follow-up (see Table 2). These costs were based on 2022 prices from the Dutch Healthcare Authority (NZA) and were reported in euros [48]. Costs for amniocentesis, CVS, and genetic follow-up testing in the pregnant woman were derived from internal rates at the Erasmus Medical Center (2022) [49], as national reimbursement costs were not specified. The actual cost of NIPT in TRIDENT-2 is uncertain, and the value of €350 may be an overestimation. Costs were not discounted due to the short evaluation period (pregnancy duration of less than one year).

**Table 2:** Cost inputs.

Unit	Base case value (€)	PSA range^a^	DSA range^a^	Reference
Pre-genetic screening counseling	76.64	±10%	±50%	[48]
Basic counseling by clinical geneticist	555.94^b^	±10%	±50%	[48]
Complex counseling by clinical geneticist	1754.02^b^	±10%	±50%	[48]
First-trimester combined test	191.23	±10%	±50%	[48]
Targeted/ genome-wide non-invasive prenatal testing	350.00^c^	±10%^c^	50–1000^c^	Assumption
Invasive testing	2654.61^d^	±10%	±50%	[48,49]
Genetic testing in pregnant woman (incl. counseling by clinical geneticist)	2761.16^e^	±10%	±50%	[48,49]
2^nd^ trimester anomaly scan (incl. counseling of the result)	166.13	±10%	±50%	[48]
Detailed diagnostic scan (incl. counseling of the result)	771.81	±10%	±50%	[48]
Biometry scan (incl. counseling of the result)	112.37	±10%	±50%	[48]

Abbreviations: DSA, deterministic sensitivity analysis; PSA, probabilistic sensitivity analysis.

^a^ In the PSA, costs were varied by ±10% around their base case values. For the deterministic sensitivity analysis, a wider range of ±50% was used to account for potential cost variations between countries.

^b^ Basic counseling for common trisomies, complex counseling for additional findings.

^c^ Estimation of NIPT costs in the TRIDENT-2 study; exact pricing remains unknown. The cost range is manually set at 50–1,000 euros, reflecting substantial differences in test costs across various countries and timeframes.

^d^ The unit cost for invasive diagnostic testing consists of obtaining fetal material by chorionic villus sampling or amniocentesis and prenatal genotyping by QF-PCR and karyotyping, and/or FISH and/or SNP array (2022).

^e^ Genetic testing in pregnant women included costs for blood sampling, karyotyping, and/or array, and counseling by a clinical geneticist.

#### Outcomes.

Outcomes analyzed included total costs of screening and diagnostics, total detected fetal chromosomal abnormalities, number of invasive procedures, and euploid fetal losses resulting from these procedures. Strategies were compared using average cost per diagnosis and incremental cost per additional diagnosed case, along with the number of invasive tests required per diagnosis. Although birth outcomes (IUFD, TOP, live births) were calculated, they were not the primary focus since the goal of prenatal screening is to detect anomalies and inform reproductive choices, and not to influence birth outcomes.

#### Model validation.

Model validation involved comparing calculated prevalence [8,13,26,50] and live birth rates [51,52] with literature estimates, validating screening outcomes with Dutch prenatal screening program data [8,30], and comparing predicted invasive test volumes with reported numbers [31].

#### Robustness of outcomes.

Probabilistic sensitivity analysis was performed to evaluate the robustness of the base-case results by varying key model parameters according to appropriate probability distributions. Probabilities were modeled using beta distributions and costs using gamma distributions, reflecting their statistical properties. Parameter ranges were primarily derived from the Dutch prenatal screening program data and existing literature. In cases where no published ranges were available, a conservative ±10% range around the base value was applied (Table 1), reflecting the reliability of the base case. The screened population size was held constant across simulations. A total of 1,000 simulations were conducted, and results were used to calculate averages and 95% confidence intervals for the total costs of the screening program, the total number of diagnosed cases, the cost per fetal case diagnosed, and the incremental cost per additional diagnosis.

One-way deterministic sensitivity analyses were conducted to assess the impact of varying individual parameters on the cost per diagnosed case across different screening strategies. This included parameters informed by TRIDENT study data and literature, as well as those expected to vary substantially across countries, such as screening uptake and healthcare costs. For these parameters, broader ranges were applied (Table 2) to enhance the analysis’s relevance to other international settings.

#### Scenario analyses.

Four scenario analyses were conducted to assess the impact of varying test uptake rates across screening strategies. The first scenario was identical to the base case except that, in the GW-NIPT strategy, women were not offered a choice between targeted and genome-wide NIPT; only GW-NIPT was available. The second scenario assumed consistent participation rates across all strategies, with the higher NIPT uptake rates applied to FCT, allowing assessment of outcome differences independent of uptake variation. The third scenario assumed 100% uptake for all screening options to estimate the theoretical upper bound of cost and detection outcomes, again without a choice in the GW-NIPT strategy. The fourth scenario limited genetic screening to women aged 36 and older, assuming a 100% uptake rate, to evaluate the economic efficiency (cost per diagnosis) when targeting older pregnant women, given the higher prevalence of chromosomal abnormalities in this group.

## Results

### Base case analysis

Table 3 summarizes the results of the base case analysis, highlighting the main outcomes of the four screening strategies, while S3 and S4 Tables provide detailed screening and birth outcomes. S1 Fig illustrates the cost per diagnosis for the different strategies and the efficient frontier.

**Table 3:** Main outcomes for the different prenatal screening strategies in a theoretical cohort of 175,000 pregnant women.

	Screening strategy			
	Second-trimester anomaly scan	FCT and second-trimester anomaly scan	Targeted NIPT and second-trimester anomaly scan	GW-NIPT and second-trimester anomaly scan
Fetal T21 diagnosed	142	272	326	326
Fetal T18 diagnosed	82	101	107	107
Fetal T13 diagnosed	32	38	41	41
Other fetal aberrations diagnosed	39	40	40	71
Total fetal common trisomies diagnosed^a^	256	411	474	474
Total fetal diagnosed cases^b^	295	452	514	545
Screened population^c^	0	61,740	78,638	78,638
Invasive tests	2,663	5,760	3,082	3,214
Euploid fetal losses^d^	3	6	3	3
Invasive tests per fetal case diagnosed	9.0	12.7	6.0	5.9
Total costs screening program (€)	52,095,591	76,862,615	81,844,411	83,115,065
Cost per screened individual (€)	–	1,245	1,041	1,057
Cost per fetal diagnosed case (€)	176,595	170,050	159,852	152,785
Incremental cost per additional fetal diagnosed case (reference strategy: scan) (€)		157,752	136,462	124,576
Incremental cost per additional fetal diagnosed case (reference strategy: FCT) (€)			81,669	67,961
Incremental cost per additional fetal diagnosed case (reference strategy: targeted NIPT) (k€)				40,989

Abbreviations: FCT, first-trimester combined test; GW, genome-wide; NIPT, non-invasive prenatal testing; T, trisomy.

^a^ Sum of all diagnosed fetal T21, T18, and T13.

^b^ Sum of all diagnosed fetal aberrations (T21, T18, T13, and the other fetal aberrations).

^c^ Screened population: pregnant women opting for FCT or NIPT. Women opting only for the second-trimester anomaly scan are not included.

^d^ Fetal losses resulting from an invasive test (chorion villus sampling or amniocentesis).

The GW-NIPT strategy detected the most fetal chromosomal abnormalities (545). Compared to second-trimester anomaly screening alone, introducing FCT increases diagnosed common trisomies by 61% (from 256 to 411), while NIPT leads to an 85% increase (from 256 to 474). Both FCT and targeted NIPT identified 40 other fetal chromosomal aberrations (additional findings) through structural abnormalities observed with the second-trimester anomaly scan, while GW-NIPT detected 31 more fetal aberrations than targeted NIPT (total of 71). Terminations of pregnancies due to diagnosed fetal chromosomal abnormalities increase by 10% with targeted NIPT (from 443 with FCT to 488) and by 18% with GW-NIPT (to 511) (S4 Table).

Implementing NIPT instead of FCT reduces the number of invasive tests, with a 46% decrease with targeted NIPT (from 5,760 to 3,082), and a 44% decrease with GW-NIPT (to 3,214), resulting in fewer euploid fetal losses. Extending NIPT analysis to the whole genome increased the total number of invasive tests by 4% from 3,082 to 3,214, because of the additional findings detected by GW-NIPT. The number of invasive tests per diagnosis decreases from ~13 with FCT to ~6 with either targeted NIPT (6.0) or GW-NIPT (5.9).

Solely screening with the second-trimester anomaly scan is the least expensive strategy (€52.1 million). Adding FCT increases costs by 48% (€76.9 million, anomalies detected: 452), targeted NIPT by 57% (€81.8 million, anomalies detected: 514), and GW-NIPT by 60% (€83.1 million, anomalies detected: 545).

When considering cost per diagnosed case, the GW-NIPT strategy was the most favorable, with €152,785 compared to €159,852 for targeted NIPT and €170,050 for FCT. The incremental cost per diagnosis versus the strategy offering only the second-trimester anomaly scan was €157,752 for FCT, €136,462 for targeted NIPT, and €124,576 for GW-NIPT. Moving from FCT to NIPT costs €81,669 for targeted NIPT and €67,961 for GW-NIPT per additional diagnosis. Switching from targeted NIPT to the GW-NIPT strategy (with a choice between targeted or genome-wide analysis) costs €40,989 per additional diagnosis.

### Model validation

The predicted prevalence of chromosomal abnormalities and live birth rates for common trisomies in the base case analysis fall within the ranges reported in the literature (S2 Fig), and the screening outcomes align with data from the Dutch prenatal screening program (S5 Table).

### Robustness of outcomes

**Fig 2:**
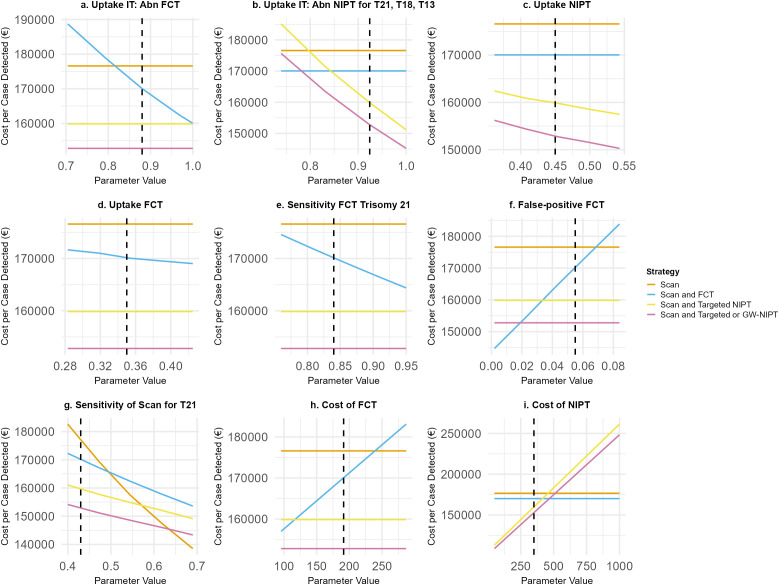
One-way deterministic sensitivity analyses showing parameters with the most impact on the outcomes. Abbreviations: Abn, abnormal; FCT, first-trimester combined testing; GW, genome-wide; IT, invasive testing; NIPT, non-invasive prenatal testing; T, trisomy; Scan, second-trimester anomaly scan. Orange: Second-trimester anomaly scan, blue: first-trimester combined testing (FCT) and second-trimester anomaly scan, yellow: targeted non-invasive prenatal testing (NIPT) and second-trimester anomaly scan, purple: Genome-wide NIPT and second-trimester anomaly scan. Base case outcomes indicated by the vertical dotted line.

Fig 2 presents results from the one-way deterministic sensitivity analysis for cost per diagnosis (additional parameters in S3 Fig).

Outcomes were most sensitive to screening uptake, invasive test uptake, and unit costs of screening tests. Because detection relies on confirmatory invasive testing, the uptake rate of invasive tests following abnormal screening results (FCT/NIPT) strongly influences results. With full uptake of invasive testing after abnormal FCT, NIPT remains the more favorable strategy on cost per diagnosis (Fig 2a). However, if invasive test uptake is 0.84 or lower for targeted NIPT or below 0.77 for GW-NIPT, FCT becomes more favorable (Fig 2b).

Screening test uptake sensitivity was examined in two ways. First, varying age-specific uptake by ±20% (Fig 2c, 2d), where higher uptake generally lowered costs without altering the relative rankings of the strategies based on cost per diagnosis. Second, varying uptake rates uniformly between 0.1 and 1 across ages showed that higher FCT uptake increases the cost per diagnosis without changing the relative ranking of the screening strategies (S3 Fig). Conversely, changes in NIPT uptake between 0.1 and 1 considerably affect the cost per diagnosis. To align with base case results, uptake rates for targeted and GW-NIPT must approach 100%, emphasizing the importance of uptake distribution across age categories, given the higher risk of chromosomal abnormalities with maternal age.

Additionally, although FCT’s sensitivity for T21 considerably affects its cost per diagnosis, the rankings remain stable within plausible ranges for this parameter (Fig 2e). However, lower FCT false-positive rates make FCT more favorable, outperforming targeted NIPT when the rate drops below *p* = 0.0325 and GW-NIPT when below *p* = 0.0184 (Fig 2f). At a sensitivity of 0.49 for T21, the second-trimester ultrasound strategy becomes preferable to FCT (Fig 2g). Finally, FCT is more favorable than targeted NIPT when its cost is below €114 (Fig 2h) or when NIPT costs exceed €418 (Fig 2i). Additionally, FCT becomes more favorable than GW-NIPT when NIPT costs exceed €467.

Probabilistic sensitivity analyses (S4 Fig) support the base-case conclusions (S6 Table). Mean number of diagnoses and total screening costs across 1,000 simulations closely matched the base case values for all strategies. In 91% of the simulations, the ranking based on the number of diagnoses was consistent with the base case. GW-NIPT most often had the lowest cost per diagnosis among strategies that include genetic screening.

### Scenario analyses

In the first scenario analysis, we evaluated the impact of removing the option between targeted and genome-wide NIPT, offering only GW-NIPT within the genome-wide strategy. Compared to the base case, this led to an increase in the number of diagnosis by 10 (from 545 to 555), due to the detection of additional chromosomal findings. Total program costs increased marginally by 0.6%, from €83.1 million to €83.6 million, while the number of invasive tests rose by 2%, from 3,214 to 3,263. The change in GW-NIPT screening test offer had no impact on the relative ranking of screening strategies based on number of diagnoses or (incremental) cost per (additional) diagnosis. Full results are presented in S7 Table.

The second scenario assessed the effect of increasing the uptake of FCT to match the higher levels observed for NIPT. As shown in S8 Table, this led to an increase in both the number of diagnosed cases and the number of invasive procedures. Specifically, the number of invasive tests per diagnosed case increased by 9% (from 12.7 to 13.9), while the cost per diagnosis rose by 4% (from €170,050 to €176,758). The incremental cost per additional diagnosis comparing FCT with targeted NIPT shifted from €81,669 to -€80,654, and for FCT versus GW-NIPT from €67,961 to -€22,913. These results indicate that higher uptake of FCT would further support the transition to NIPT as the more favorable screening strategy.

In the third scenario, we assumed 100% uptake of all available screening options to assess the upper bounds of diagnostic yield and program costs (S9 Table). In this scenario, the total screening program costs were highest for FCT (€127.8 million), followed by GW-NIPT (€127.4 million), targeted NIPT (€123.9 million), and second-trimester ultrasound only (€58.8 million). The cost per diagnosis decreased slightly for most strategies, from €176,595 to €169,361 for second-trimester ultrasound scan only, from €159,852 to €159,026 for targeted NIPT, and more substantially for GW-NIPT from €152,785 to €146,271. Conversely, it increased for FCT, from €170,050 to €184,402. The incremental cost per additional diagnosis comparing targeted NIPT and GW-NIPT with FCT also changed, from €81,669 to -€45,454, and from €67,961 to -€2,171, respectively.

Finally, in the fourth scenario, screening was limited to women aged 36 years and older, assuming 100% uptake within this subgroup (S10 Table). Compared to the third scenario, this resulted in an 81% reduction in the screened population across all strategies. Correspondingly, program costs decreased by 43% for FCT (from €127.8 million to €72.6 million), 42% for targeted NIPT (from €123.9 million to €72.0 million), and 43% for GW-NIPT (from €127.4 million to €72.7 million). Despite the smaller screened population, the number of diagnosis declined to a lesser extent due to the higher prevalence of chromosomal abnormalities in this age group: 20% reduction for FCT (693 to 556), 22% for targeted NIPT (779 to 609), and 29% for GW-NIPT (872 to 623). GW-NIPT remained the most favorable on cost per diagnosis in this restricted population, with a cost per diagnosis of €130,196 for FCT, €118,014 for targeted NIPT, and €116,431 for GW-NIPT.

## Discussion

Our study shows that offering NIPT to the general obstetric population, with a choice between GW-NIPT and targeted NIPT, improves detection of fetal chromosomal abnormalities relative to other prenatal screening strategies. Relative to FCT, this approach increases detection rates by ~20% and reduces invasive testing by ~45%. It also yields the lowest cost per diagnosed case (€152,785). Expanding from targeted NIPT to GW-NIPT adds 6% in diagnoses at €40,989 per additional case. These findings support the adoption of GW-NIPT based on its diagnostic yield and cost efficiency. Because no recognized threshold exists for interpreting cost per anomaly detected, these findings should be interpreted as relative clinical and economic comparisons, not as absolute cost-effectiveness claims.

Our analysis underscores the clinical value of offering GW-NIPT alongside targeted NIPT in prenatal screening. Compared to FCT, screening with GW-NIPT considerably increases detection rates for fetal chromosomal abnormalities while reducing the number of invasive procedures, thereby minimizing the risk of procedure-related miscarriages. By enhancing the detection of chromosomal anomalies and reducing invasive testing, GW-NIPT provides a comprehensive approach that better supports reproductive autonomy.

Generalizability of the results was assessed through extensive sensitivity analyses, revealing robust findings across a wide range of values, thus increasing the relevance of our study in various contexts. The outcomes were most influenced by the costs of FCT and NIPT, as well as the uptake rates of NIPT and subsequent invasive testing following abnormal results. Despite variations in these factors, the ranking of the screening scenarios remained largely stable.

Our scenario analyses showed that program design choices can meaningfully impact cost, diagnostic yield, and economic efficiency. Restricting screening to women aged 36 and older substantially reduced overall costs, with a relatively smaller decline in diagnoses due to higher anomaly prevalence in this group. While this age-based approach may be a feasible option in resource-constrained settings or as a phased introduction, it would exclude a large cohort of younger women who could still benefit from the detection of fetal chromosomal abnormalities at a reasonable cost per diagnosis. Conversely, increasing uptake of FCT raised its cost per diagnosis, further supporting the transition to NIPT. Offering only GW-NIPT, as opposed to the base case scenario in which a choice was offered between targeted NIPT and GW-NIPT, slightly increased diagnoses and costs without altering the ranking of strategies. Full uptake scenarios demonstrated the upper bounds of program cost and diagnostic yield, and confirmed that NIPT strategies remain the most favorable on cost per diagnosis under a range of assumptions.

In 2023, the Dutch Health Council revised its guidance on the reporting of additional findings, recommending that only SAs should be reported following the full integration of NIPT into the prenatal screening program [8,53]. The committee concluded that the potential disadvantages of reporting RATs outweighed the benefits. This recommendation was subsequently adopted and translated into policy. GW-NIPT became the standard first-tier test in the program as of April 1, 2023, and the reporting of RATs was discontinued as of April 2025. As RATs constitute a small share of fetal chromosomal abnormalities, we expect minimal impact on our conclusions.

One of the main strengths of this study is the use of data from the unique TRIDENT-2 study and the national screening database, Peridos, providing a solid evidence base for important model parameters. Additionally, our study incorporated the second-trimester ultrasound scan, which is part of the Dutch national prenatal screening program and a routine component of prenatal care in nearly all high-income countries [18,19]. Including this scan in clinical and economic impact analysis of genetic prenatal screening tests is essential, as it identifies structural anomalies with potential genetic origins. In clinical practice, detection of such anomalies often triggers follow-up testing, including invasive procedures. Excluding it would overstate the added value of genetic screening strategies by not accounting for diagnoses already made during routine care. This approach allowed us to demonstrate a realistic estimate of the incremental value of NIPT beyond routine ultrasound.

Despite its strengths, this study has several limitations. Most notably, we did not conduct a conventional cost-effectiveness analysis using metrics like cost per QALY gained. In prenatal screening, such measures present considerable ethical and methodological difficulties. The goal is to inform reproductive choices rather than to improve health outcomes directly, and quantifying outcomes like pregnancy termination or preparing for an affected child using QALYs is inherently problematic. It also remains unclear whose QALYs should be considered, and the diversity of genetic conditions further complicates valuation. In addition, reliable long-term cost data are often unavailable. For these reasons, we focused on cost per case detected and incremental cost per additional diagnosis, which are metrics commonly used in economic evaluations of prenatal screening programs and aligned with the primary goal of enabling informed reproductive choices [20]. While this limits comparability across broader health interventions, it avoids ethically challenging assumptions and reflects standard practice in this domain.

Another limitation is the exclusion of twin pregnancies. Although the incidence of twin pregnancies is rising, partly due to increasing maternal age and an increasing use of assisted reproduction technologies, they still represent only a small fraction of pregnancies in the Netherlands (approximately 1.5%) [27]. Furthermore, the performance characteristics of NIPT in twins, particularly for conditions other than trisomy 21, remain less well established due to limited data [54,55]. Including them would have introduced substantial uncertainty in key model parameters. We therefore chose to exclude twin pregnancies in order to preserve the reliability and robustness of the model. Additionally, the model did not account for the one-time implementation costs of NIPT, such as laboratory setup, which could affect feasibility assessments in different healthcare systems. Lastly, although GW-NIPT offers broader detection capabilities, it also increases the likelihood of identifying variants of unknown significance and detecting maternal and placental abnormalities due to the nature of the tested material. This may induce anxiety, which is not fully captured in current outcome measures. This underscores the need for careful pretest counseling and consideration of the psychological impact of more comprehensive testing before integrating GW-NIPT into screening programs [21,22].

Our study adds to the growing body of evidence supporting the clinical benefits and cost considerations of incorporating NIPT into prenatal screening programs. While previous studies have focused primarily on targeted NIPT and/or contingent NIPT (used after initial high-risk screening results) [13,14,50] or expanded panels for specific chromosomal anomalies [15–17], our analysis evaluates the broader implementation of GW-NIPT. By demonstrating that GW-NIPT offers the highest diagnostic yield and the most favorable cost per diagnosis among strategies assessed, our study provides strong support for its adoption as a routine first-tier screening test. This conclusion challenges current practices that rely on contingent or targeted NIPT, or FCT. While NIPT offers better clinical and economic outcomes, its broad implementation requires careful consideration of economic, societal, and ethical factors [15,16].

Lastly, the Netherlands is one of the few countries that routinely offers NIPT to the general obstetric population. While NIPT has shown to increase detection rates and decrease unnecessary invasive procedures and related miscarriages, its higher costs and the investments needed for national implementation may be challenging for some countries. In the Dutch context, however, national integration has substantially reduced the cost of NIPT, bringing it well below the €350 estimated in this study, although precise figures are not publicly available. This underscores the potential benefits of national integration in improving the cost per diagnosis by NIPT.

In conclusion, both screening strategies, offering targeted or GW-NIPT, detect more fetal chromosomal aberrations than FCT, thereby enhancing reproductive autonomy for prospective parents. GW-NIPT provides the greatest diagnostic yield and the most favorable cost per diagnosis, justifying its implementation in prenatal screening programs. To build on our observations, future research should explore the integration of GW-NIPT with emerging first-trimester screening protocols, including the newly introduced first-trimester anomaly scan [56]. Evaluations of combined approaches could help optimize detection rates and improve economic efficiency. Additionally, longitudinal studies are needed to examine the long-term outcomes of pregnancies following GW-NIPT, particularly in terms of healthcare utilization, cost savings from reduced invasive procedures, and psychosocial impact on families, which may also make it possible to conduct a formal cost-effectiveness analysis.

## Supporting information

S1 Table Population distribution (%) and prevalence of chromosomal abnormalities (%) from age ≤ 15 to  ≥ 49 at 12-week gestation. (DOCX)

S2 Table Screening uptake (%) according to maternal age. (DOCX)

S3 Table Detailed screening outcomes for the four screening strategies—base case. (DOCX)

S4 Table Detailed birth outcomes for the four screening strategies—base case. (DOCX)

S5 Table Validation of modeled screening outcomes and invasive tests performed. (DOCX)

S6 Table Probabilistic sensitivity analyses output. (DOCX)

S7 Table Main outcomes of scenario analysis 1, which is identical to the base case except that, in the GW-NIPT strategy, women were not offered a choice between targeted and genome-wide NIPT; only GW-NIPT is available. (DOCX)

S8 Table Main outcomes of scenario analysis 2, assuming consistent participation rates across all strategies, with the higher NIPT uptake rates applied to FCT. (DOCX)

S9 Table Main outcomes of scenario analysis 3, assuming 100% uptake for all screening options, without a choice in the GW-NIPT strategy (only GW-NIPT is available). (DOCX)

S10 Table Main outcomes of scenario analysis 4, presuming only women of 36 years old or older were invited to genetic screening and assuming 100% of this population opted for FCT or NIPT screening. (DOCX)

S1 Fig Clinical and economic outcomes in the base case analysis. Abbreviations: FCT, first-trimester combined test; GW, genome-wide; NIPT, non-invasive prenatal testing; scan, second-trimester anomaly scan. In this Figure, each point represents a different prenatal screening strategy with its associated cost and number of fetal chromosomal aberrations detected. The efficient frontier is the boundary that divides the plane into regions of optimal and suboptimal choices. The strategies on the efficient frontier represent interventions that provide the maximum number of fetal chromosomal aberrations detected for a given cost or the minimum cost for a given level of diagnoses. Interventions above or to the left of this efficient frontier are considered dominated because they detect a lower number of fetal chromosomal abnormalities for higher costs or higher costs for the same number of cases detected. The efficient frontier thus identifies the set of interventions that optimize the trade-off between cost and diagnoses, helping decision-makers towards the most efficient allocation of resources.
(TIF)

S2 Fig Validation of modeled prevalence of chromosomal aberrations and live birth prevalence of common trisomies 21, 18, and, 13. The dot shows the modeled value. The line indicates the range from the minimum to maximum parameter values based on the literature. The prevalence of autosomal trisomies and structural aberrations is based on TRIDENT-2 data and was assumed in the model; therefore, the min–max line coincides with the modeled parameter value.
(TIF)

S3 Fig One-way deterministic sensitivity analysis of model parameters. Abbreviations: FCT, first-trimester combined testing; FU, follow-up; GW, genome-wide; IT, invasive testing; NIPT, non-invasive prenatal testing; RAT, rare autosomal trisomy; SA, structural aberration; scan, second-trimester anomaly scan; T, trisomy. Orange: Second-trimester anomaly scan, Blue: first-trimester combined testing (FCT) and second-trimester anomaly scan, Yellow: Targeted non-invasive prenatal testing (NIPT) and second-trimester anomaly scan, Purple: Genome-wide NIPT and second-trimester anomaly scan. Base case outcomes indicated by the vertical dotted line.
(TIF)

S4 Fig Probabilistic sensitivity analysis (1,000 simulations): cost-diagnosis scatter plot. Abbreviations: FCT, first-trimester combined testing; GW, genome-wide; NIPT, non-invasive prenatal testing; scan, second-trimester anomaly scan. Orange: Second-trimester anomaly scan, Blue: first-trimester combined testing (FCT) and second-trimester anomaly scan, Yellow: Targeted non-invasive prenatal testing (NIPT) and second-trimester anomaly scan, Purple: GW-NIPT and second-trimester anomaly scan. The colored dot with a black outline represents each strategy’s mean cost and cases diagnosed. The dotted ellipse indicates the 95% bivariate confidence interval. The vertical and horizontal lines through the mean show the univariate 95% confidence interval for costs and diagnoses.
(TIF)

S1 Checklist CHEERS 2022 checklist. The checklist is Open Access distributed in accordance with the terms of the Creative Commons Attribution (CC BY 4.0) license.
(DOCX)

## Abbreviations

cfDNA: cell-free DNA
CPM: confined placental mosaicism
CVS: chorionic villus sampling
FCT: first-trimester combined test
GW-NIPT: genome-wide non-invasive prenatal testing
IM: iatrogenic miscarriage
IUFD: intrauterine fetal demise
QALY: quality-adjusted life year
RAT: rare autosomal trisomy
SA: structural aberration
TOP: termination of pregnancy
TRIDENT-2: TRIal by Dutch laboratories for Evaluation of Non-invasive prenatal Testing
