# Supplementary material for: Clinical and economic impact of genome-wide non-invasive prenatal testing (NIPT) as a first-tier screening method compared to targeted NIPT and first-trimester combined testing: A modeling study
Source: PLoS Med. 2025 Nov 5;22(11):e1004790. doi: 10.1371/journal.pmed.1004790 (PMC12611151; doi:10.1371/journal.pmed.1004790)
Supplement: S1 Table — (DOCX) [file pmed.1004790.s001.docx]

**S1 Table.** Population distribution (%) and prevalence of chromosomal abnormalities (%) from age ≤15 - ≥49 at 12-week gestation

| Maternal  age | % of total  pregnancies^a^ | Trisomy 21 prevalence (%)^b^ | Trisomy 18  prevalence (%)^b^ | Trisomy 13  prevalence (%)^b^ | Rare autosomal trisomy  prevalence (%)^c^ | Structural aberration  prevalence (%)^c^ |
| --- | --- | --- | --- | --- | --- | --- |
| ≤15 | 0.0056 | 0.0368 | 0.0120 | 0.0000 | 0.1800 | 0.1700 |
| 16 | 0.0183 | 0.0368 | 0.0120 | 0.0000 | 0.1800 | 0.1700 |
| 17 | 0.0596 | 0.0368 | 0.0120 | 0.0000 | 0.1800 | 0.1700 |
| 18 | 0.1371 | 0.0368 | 0.0120 | 0.0000 | 0.1800 | 0.1700 |
| 19 | 0.3314 | 0.0368 | 0.0120 | 0.0000 | 0.1800 | 0.1700 |
| 20 | 0.5081 | 0.0368 | 0.0120 | 0.0000 | 0.1800 | 0.1700 |
| 21 | 0.7908 | 0.0840 | 0.0280 | 0.0000 | 0.1800 | 0.1700 |
| 22 | 1.1408 | 0.0575 | 0.0140 | 0.0140 | 0.1800 | 0.1700 |
| 23 | 1.6866 | 0.0983 | 0.0110 | 0.0110 | 0.1800 | 0.1700 |
| 24 | 2.3539 | 0.0801 | 0.0340 | 0.0080 | 0.1800 | 0.1700 |
| 25 | 3.2432 | 0.0903 | 0.0200 | 0.0100 | 0.1800 | 0.1700 |
| 26 | 4.2328 | 0.0881 | 0.0170 | 0.0170 | 0.1800 | 0.1700 |
| 27 | 5.4025 | 0.1169 | 0.0220 | 0.0100 | 0.1800 | 0.1700 |
| 28 | 6.6191 | 0.0942 | 0.0260 | 0.0210 | 0.1800 | 0.1700 |
| 29 | 7.4837 | 0.1067 | 0.0260 | 0.0240 | 0.1800 | 0.1700 |
| 30 | 8.2429 | 0.1240 | 0.0400 | 0.0160 | 0.1800 | 0.1700 |
| 31 | 8.6882 | 0.1511 | 0.0310 | 0.0190 | 0.1800 | 0.1700 |
| 32 | 8.6392 | 0.1568 | 0.0610 | 0.0290 | 0.1800 | 0.1700 |
| 33 | 8.0300 | 0.2348 | 0.0430 | 0.0080 | 0.1800 | 0.1700 |
| 34 | 6.9845 | 0.2839 | 0.0550 | 0.0260 | 0.1800 | 0.1700 |
| 35 | 6.0937 | 0.3452 | 0.0830 | 0.0310 | 0.1800 | 0.1700 |
| 36 | 5.0861 | 0.4295 | 0.0860 | 0.0520 | 0.1800 | 0.1700 |
| 37 | 4.0523 | 0.6853 | 0.0990 | 0.0770 | 0.1800 | 0.1700 |
| 38 | 3.2019 | 0.8464 | 0.2110 | 0.0990 | 0.1800 | 0.1700 |
| 39 | 2.4008 | 1.1469 | 0.3080 | 0.0970 | 0.1800 | 0.1700 |
| 40 | 1.7435 | 1.3544 | 0.4230 | 0.1270 | 0.1800 | 0.1700 |
| 41 | 1.1677 | 2.3503 | 0.6670 | 0.0920 | 0.1800 | 0.1700 |
| 42 | 0.7248 | 2.6421 | 1.1380 | 0.0000 | 0.1800 | 0.1700 |
| 43 | 0.4290 | 3.2799 | 1.2460 | 0.0000 | 0.1800 | 0.1700 |
| 44 | 0.2203 | 2.3774 | 1.3510 | 0.1520 | 0.1800 | 0.1700 |
| 45 | 0.1215 | 3.2520 | 1.3810 | 0.2790 | 0.1800 | 0.1700 |
| 46 | 0.0705 | 3.2520 | 1.3810 | 0.2790 | 0.1800 | 0.1700 |
| 47 | 0.0363 | 3.2520 | 1.3810 | 0.2790 | 0.1800 | 0.1700 |
| 48 | 0.0177 | 3.2520 | 1.3810 | 0.2790 | 0.1800 | 0.1700 |
| ≥49 | 0.0357 | 3.2520 | 1.3810 | 0.2790 | 0.1800 | 0.1700 |

*Abbreviations: FCT, first trimester combined testing; NIPT, non-invasive prenatal testing. ^a^Statistics Netherlands 2018-2019^1^
^b^Hartwig 2016^2^
^c^ van Prooyen Schuurman 2022.^3^ Of the 0.18 rare autosomal trisomies, 8% confirmed in the fetus, 91% from the placenta (i.e. confined placental mosaicism) and 1% from the mother. Of the 0.17% structural aberrations this is 44%, 6%, and 50% respectively.*

**References**

1. Centraal Bureau voor de Statistiek (CBS). (2019). Geboorte. *1 januari 2024*. https://opendata.cbs.nl

2. Hartwig. T.S.. Sorensen. S.. and Jorgensen. F.S. (2016). The maternal age-related first trimester risks for trisomy 21. 18 and 13 based on Danish first trimester data from 2005 to 2014. Prenat Diagn *36*. 643-649. 10.1002/pd.4833.

3. van Prooyen Schuurman. L.. Sistermans. E.A.. Van Opstal. D.. Henneman. L.. Bekker. M.N.. Bax. C.J.. Pieters. M.J.. Bouman. K.. de Munnik. S.. den Hollander. N.S.. et al. (2022). Clinical impact of additional findings detected by genome-wide non-invasive prenatal testing: Follow-up results of the TRIDENT-2 study. Am J Hum Genet *109*. 1140-1152. 10.1016/j.ajhg.2022.04.018.
