# Supplementary material for: Clinical and economic impact of genome-wide non-invasive prenatal testing (NIPT) as a first-tier screening method compared to targeted NIPT and first-trimester combined testing: A modeling study
Source: PLoS Med. 2025 Nov 5;22(11):e1004790. doi: 10.1371/journal.pmed.1004790 (PMC12611151; doi:10.1371/journal.pmed.1004790)
Supplement: S2 Table — (DOCX) [file pmed.1004790.s002.docx]

**S2 Table.** Screening uptake (%) according to maternal age

| Maternal age | Strategy 2: Uptake FCT^a^ | Strategy 3 (solely targeted screening): Uptake targeted NIPT^b^ | Strategy 4 (targeted or genome-wide screening): Uptake targeted NIPT | Strategy 4 (targeted or genome-wide screening): Uptake GW-NIPT^b^ |
| --- | --- | --- | --- | --- |
| ≤25 | 14.2 | 21.8 | 6.2 | 15.7 |
| 26-30 | 28.1 | 41.6 | 11.6 | 30.0 |
| 31-35 | 39.8 | 51.2 | 12.8 | 38.3 |
| 36-40 | 50.9 | 53.2 | 12.3 | 40.9 |
| ≥41 | 46.0 | 48.9 | 10.7 | 38.3 |
| Total | 33.0 | 43.9 | 11.4 | 32.6 |

*Abbreviations: FCT, first trimester combined testing; GW, genome-wide; NIPT, non-invasive prenatal testing.
^a^Calculated over 2014-2016 by dividing the registered performed FCTs by the total number of singleton pregnancies in the same period (Peridos) .^1^
^b^Calculated over 2018-2019 by dividing the registered performed NIPTs by the total number of singleton pregnancies in the same period (Peridos).^1^*

**References**

1. Online national digital registration system for prenatal screening Peridos. <https://www.peridos.nl>.
