# Supplementary material for: Clinical and economic impact of genome-wide non-invasive prenatal testing (NIPT) as a first-tier screening method compared to targeted NIPT and first-trimester combined testing: A modeling study
Source: PLoS Med. 2025 Nov 5;22(11):e1004790. doi: 10.1371/journal.pmed.1004790 (PMC12611151; doi:10.1371/journal.pmed.1004790)
Supplement: S3 Table — (DOCX) [file pmed.1004790.s003.docx]

**S3 Table.** Detailed screening outcomes for the four screening strategies – base case

|  |  | Screening strategy | | | | | | | |
| --- | --- | --- | --- | --- | --- | --- | --- | --- | --- |
|  |  | Second trimester anomaly scan | | FCT and second trimester anomaly scan | | Targeted NIPT and second trimester anomaly scan | | GW-NIPT and second trimester anomaly scan | |
| Cases of fetal chromosomal aberrations |  | Common  Trisomies | Additional  Findings | Common  Trisomies | Additional  Findings | Common  Trisomies | Additional  Findings | Common  Trisomies | Additional  Findings |
| No test^a^ |  | 122 | 24 | 103 | 23 | 109 | 23 | 109 | 23 |
| Missed^b^ | *Scan* | 291 | 73 | 149 | 43 | 127 | 35 | 127 | 35 |
|  | *Screen & scan* | 0 | 0 | 24 | 29 | 2 | 39 | 2 | 10 |
| Suspected^c^ | *Scan* | 130 | 20 | 76 | 19 | 58 | 20 | 58 | 12 |
|  | *Screen* | 0 | 0 | 14 | 0 | 12 | 0 | 12 | 3 |
|  | *Screen & scan* | 0 | 0 | 21 | 0 | 18 | 0 | 18 | 3 |
| Diagnosed^d^ | *It direct* | 3 | 1 | 3 | 1 | 4 | 1 | 4 | 1 |
|  | *Scan* | 252 | 39 | 147 | 38 | 112 | 39 | 112 | 23 |
|  | *Screen* | 0 | 0 | 260 | 3 | 357 | 0 | 357 | 46 |

*Abbreviations: FCT, first trimester combined testing; GW, genome-wide; NIPT, non-invasive prenatal testing. ^a^No test: no second-trimester anomaly scan/FCT/NIPT/invasive diagnostic testing performed
^b^Missed: false-negative second-trimester anomaly scan or advanced ultrasound/ false-negative second-trimester anomaly scan or advanced ultrasound, and/or false negative screening test (FCT/ NIPT). ‘Missed’ means that a fetal chromosomal aberration was present but not detected by a scan or genetic screening test. In the case of an ultrasound, this means either no structural abnormalities were present for the sonographer to identify, or a structural abnormality was present but the sonographer missed it.
^c^Suspected: screen-positive second-trimester anomaly scan and/or screening test (FCT/NIPT), without confirmation by invasive diagnostic testing, due to rejection of invasive procedures.
^d^Diagnosed: Chromosomal aberration confirmed by invasive diagnostic testing in pregnancies where parents directly opted for invasive testing without prior screening, or following a screen-positive second-trimester anomaly scan or screening test (FCT/NIPT)*
