# Supplementary material for: Clinical and economic impact of genome-wide non-invasive prenatal testing (NIPT) as a first-tier screening method compared to targeted NIPT and first-trimester combined testing: A modeling study
Source: PLoS Med. 2025 Nov 5;22(11):e1004790. doi: 10.1371/journal.pmed.1004790 (PMC12611151; doi:10.1371/journal.pmed.1004790)
Supplement: S4 Table — (DOCX) [file pmed.1004790.s004.docx]

**S4 Table.** Detailed birth outcomes for the four screening strategies – base case

|  | Screening strategy | | | | | | | |
| --- | --- | --- | --- | --- | --- | --- | --- | --- |
| Cases of fetal chromosomal aberrations | Second trimester anomaly scan | | FCT &  second trimester anomaly scan | | Targeted NIPT & second trimester anomaly scan | | GW-NIPT & second trimester anomaly scan | |
|  | Common  Trisomies | Additional  Findings | Common  Trisomies | Additional  Findings | Common  Trisomies | Additional  Findings | Common  Trisomies | Additional  Findings |
| *Iatrogenic miscarriage* | 0 | 0 | 0 | 0 | 1 | 0 | 1 | 0 |
| *Termination of pregnancy* | 276 | 42 | 399 | 44 | 445 | 43 | 445 | 66 |
| *Intra-uterine fetal demise* | 223 | 3 | 172 | 3 | 154 | 3 | 154 | 3 |
| *Live birth* | 298 | 111 | 226 | 109 | 198 | 110 | 198 | 88 |

*Abbreviations: FCT, first trimester combined testing, GW, genome-wide; NIPT, non-invasive prenatal testing.*
