# Supplementary material for: Clinical and economic impact of genome-wide non-invasive prenatal testing (NIPT) as a first-tier screening method compared to targeted NIPT and first-trimester combined testing: A modeling study
Source: PLoS Med. 2025 Nov 5;22(11):e1004790. doi: 10.1371/journal.pmed.1004790 (PMC12611151; doi:10.1371/journal.pmed.1004790)
Supplement: S5 Table — (DOCX) [file pmed.1004790.s005.docx]

**S5 Table.** Validation of modelled screening outcomes and invasive tests performed

|  | Validation target | Modelled value |
| --- | --- | --- |
| Cases with a suspicion of an anomaly on the second-trimester anomaly scan | 0.044%^3^ | 0.047% |
| Cases with structural aberration confirmed by advanced scan after second-trimester scan | 0.38%^3^ | 0.42% |
| Uptake FCT | 0.330%^2^ | 0.338% |
| Total increased risk for Trisomy 21, Trisomy 18, and Trisomy 13 with FCT | 0.059%^3^ | 0.062% |
| Confirmed fetal aberrations following an abnormal FCT | 0.0014%^3^ | 0.0015% |
| Confirmed Trisomy 21 per 10.000 GW- NIPT | 33^4^ | 33.1 |
| Confirmed Trisomy 18 per 10.000 GW-NIPT | 8^4^ | 8.9 |
| Confirmed Trisomy 13 per 10.000 GW-NIPT | 4^4^ | 3.4 |
| Confirmed other fetal aberrations per 10.000 genome-wide NIPT | 7^1^ | 5.9 |
| Percentage of all pregnancies with an invasive test for suspicion of an anomaly on second trimester ultrasound | 0.95%^5^ | 1.01% |
| Percentage of all pregnancies with an invasive test for abnormal FCT | 1.72%^5^ | 1.85% |
| Percentage of all pregnancies with an invasive test for abnormal GW-NIPT | 0.28%^5^ | 0.31% |
| Percentage of all pregnancies with an invasive test for other reason | 0.43%^5^ | 0.43% |

*Abbreviations: FTC, first trimester combined testing; GW, genome-wide; NIPT, non-invasive prenatal testing.*

**SUPPLEMENTAL REFERENCES**

1. van Prooyen Schuurman. L.. Sistermans. E.A.. Van Opstal. D.. Henneman. L.. Bekker. M.N.. Bax. C.J.. Pieters. M.J.. Bouman. K.. de Munnik. S.. den Hollander. N.S.. et al. (2022). Clinical impact of additional findings detected by genome-wide non-invasive prenatal testing: Follow-up results of the TRIDENT-2 study. Am J Hum Genet *109*. 1140-1152. 10.1016/j.ajhg.2022.04.018.

2. Online national digital registration system for prenatal screening Peridos. <https://www.peridos.nl>.

3. Scientific Center for Quality of Healthcare (IQ healthcare). Professionalsmonitor 2014-2020 Prenatale screening op down-. edwards- en patausyndroom en het Structureel Echoscopisch Onderzoek.

4. van der Meij. K.R.M.. Sistermans. E.A.. Macville. M.V.E.. Stevens. S.J.C.. Bax. C.J.. Bekker. M.N.. Bilardo. C.M.. Boon. E.M.J.. Boter. M.. Diderich. K.E.M.. et al. (2019). TRIDENT-2: National Implementation of Genome-wide Non-invasive Prenatal Testing as a First-Tier Screening Test in the Netherlands. Am J Hum Genet *105*. 1091-1101. 10.1016/j.ajhg.2019.10.005.

5. Working Group on Prenatal Diagnostics. (2012-2019). Annual Reports.
