# Supplementary material for: Clinical and economic impact of genome-wide non-invasive prenatal testing (NIPT) as a first-tier screening method compared to targeted NIPT and first-trimester combined testing: A modeling study
Source: PLoS Med. 2025 Nov 5;22(11):e1004790. doi: 10.1371/journal.pmed.1004790 (PMC12611151; doi:10.1371/journal.pmed.1004790)
Supplement: S6 Table — (DOCX) [file pmed.1004790.s006.docx]

**S6 Table.** Probabilistic sensitivity analyses output

| Probabilistic sensitivity analyses output^a^ ↓ | Screening strategy | | | |
| --- | --- | --- | --- | --- |
|  | Second trimester anomaly scan | FCT &  second trimester anomaly scan | Targeted NIPT & second trimester anomaly scan | GW-NIPT & second trimester anomaly scan |
| Total costs screening program (M€) [95% CI] | 52.17 [46.10;58.81] | 76.59 [63.01;94.51] | 81.90 [72.66;91.69] | 83.18 [73.86;93.12] |
| Total fetal diagnosed cases [95% CI] | 298 [201;400] | 454 [372;530] | 516 [408;592] | 547 [444;625] |
| Cost per fetal diagnosed case (€) [95% CI] | 180,304 [129,195;260,967] | 169,813 [135,469;219,867] | 160,140 [134,641;199,226] | 153,175 [129,494;186,878] |
| Incremental cost per additional fetal diagnosed case (ref strategy: scan) (€) [95% CI] |  | 163,531  [82,550;301,556] | 143,711  [95,781;256,207] | 134,997 [89,827;208,977] |
| Incremental cost per additional fetal diagnosed case (ref strategy: FCT) (€) [95% CI] |  |  | 81,378  [-518,682;983,632] | 108,404  [-269,287;485,967] |
| Incremental cost per additional fetal diagnosed case (ref strategy: targeted NIPT) (€) [95% CI] |  |  |  | 41,684  [32,652;60,089] |

*Abbreviations: CI, Confidence Interval; FCT, first trimester combined testing; GW, genome-wide; NIPT, non-invasive prenatal testing.
^a^Average values over 1000 simulations*
