# Supplementary material for: Clinical and economic impact of genome-wide non-invasive prenatal testing (NIPT) as a first-tier screening method compared to targeted NIPT and first-trimester combined testing: A modeling study
Source: PLoS Med. 2025 Nov 5;22(11):e1004790. doi: 10.1371/journal.pmed.1004790 (PMC12611151; doi:10.1371/journal.pmed.1004790)
Supplement: S7 Table — (DOCX) [file pmed.1004790.s007.docx]

**S7 Table.** Main outcomes of scenario analysis 1, which is identical to the base case except that, in the GW-NIPT strategy, women were not offered a choice between targeted and genome-wide NIPT; only GW-NIPT is available

|  | Screening strategy | | | |
| --- | --- | --- | --- | --- |
|  | Second trimester anomaly scan | FCT &  second trimester anomaly scan | Targeted NIPT & second trimester anomaly scan | GW-NIPT & second trimester anomaly scan |
| Fetal T21 diagnosed | 142 | 272 | 326 | 326 |
| Fetal T18 diagnosed | 82 | 101 | 107 | 107 |
| Fetal T13 diagnosed | 32 | 38 | 41 | 41 |
| Other fetal aberrations diagnosed | 39 | 40 | 40 | 81 |
| Total fetal common trisomies diagnosed^a^ | 256 | 411 | 474 | 474 |
| Total fetal diagnosed cases^b^ | 295 | 452 | 514 | 555 |
| Screened population^c^ | 0 | 61,740 | 78,638 | 78,638 |
| Invasive tests | 2,663 | 5,760 | 3,082 | 3,263 |
| Euploid fetal losses^d^ | 3 | 6 | 3 | 3 |
| Invasive tests per fetal case diagnosed | 9.0 | 12.7 | 6.0 | 5.9 |
| Total costs screening program (€) | 52,095,591 | 76,862,615 | 81,844,411 | 83,625,580 |
| Cost per screened individual (€) | - | 1,245 | 1,041 | 1,063 |
| Cost per fetal diagnosed case (€) | 176,595 | 170,050 | 159,852 | 150,677 |
| Incremental cost per additional fetal diagnosed case (ref strategy: scan) (€) |  | 157,752 | 136,462 | 121,269 |
| Incremental cost per additional fetal diagnosed case (ref strategy: FCT) (€) |  |  | 81,669 | 65,660 |
| Incremental cost per additional fetal diagnosed case (ref strategy: targeted NIPT) (€) |  |  |  | 43,443 |

*Abbreviations: FCT. first-trimester combined test; GW. genome-wide; NIPT. non-invasive prenatal testing; T. trisomy.
^a^Sum of all diagnosed fetal T21. T18. and T13
^b^Sum of all diagnosed fetal aberrations (T21. T18. T13. and the other fetal aberrations) ^c^Screened population: pregnant women opting for FCT or NIPT.* *Women opting only for the second trimester anomaly scan are not included. ^d^Fetal losses resulting from an invasive test (chorion villus sampling or amniocentesis)*
