# Supplementary material for: Clinical and economic impact of genome-wide non-invasive prenatal testing (NIPT) as a first-tier screening method compared to targeted NIPT and first-trimester combined testing: A modeling study
Source: PLoS Med. 2025 Nov 5;22(11):e1004790. doi: 10.1371/journal.pmed.1004790 (PMC12611151; doi:10.1371/journal.pmed.1004790)
Supplement: S8 Table — (DOCX) [file pmed.1004790.s008.docx]

**S8 Table.** Main outcomes of scenario analysis 2, assuming consistent participation rates across all strategies with the higher NIPT uptake rates applied to FCT

|  | Screening strategy | | | |
| --- | --- | --- | --- | --- |
|  | Second trimester anomaly scan | FCT &  second trimester anomaly scan | Targeted NIPT & second trimester anomaly scan | GW-NIPT & second trimester anomaly scan |
| Fetal T21 diagnosed | 142 | 292 | 326 | 326 |
| Fetal T18 diagnosed | 82 | 104 | 107 | 107 |
| Fetal T13 diagnosed | 32 | 40 | 41 | 41 |
| Other fetal aberrations diagnosed | 39 | 43 | 40 | 71 |
| Total fetal common trisomies diagnosed^a^ | 256 | 436 | 474 | 474 |
| Total fetal diagnosed cases^b^ | 295 | 479 | 514 | 545 |
| Screened population^c^ | 0 | 79,062 | 78,638 | 78,638 |
| Invasive tests | 2,663 | 6,655 | 3,082 | 3,214 |
| Euploid fetal losses^d^ | 3 | 7 | 3 | 3 |
| Invasive tests per fetal case diagnosed | 9.0 | 13.9 | 6.0 | 5.9 |
| Total costs screening program (€) | 52,095,591 | 84,667,310 | 81,844,411 | 83,155,065 |
| Cost per screened individual (€) | - | 1,071 | 1,041 | 1,057 |
| Cost per fetal diagnosed case (€) | 176,595 | 176,758 | 159,852 | 152,785 |
| Incremental cost per additional fetal diagnosed case (ref strategy: scan) (€) |  | 177,020 | 136,462 | 124,576 |
| Incremental cost per additional fetal diagnosed case (ref strategy: FCT) (€) |  |  | -80,654 | -22,913 |
| Incremental cost per additional fetal diagnosed case (ref strategy: targeted NIPT) (€) |  |  |  | 40,989 |

*Abbreviations: FCT. first-trimester combined test; GW. genome-wide; NIPT. non-invasive prenatal testing; T. trisomy.
^a^Sum of all diagnosed fetal T21. T18. and T13
^b^Sum of all diagnosed fetal aberrations (T21. T18. T13. and the other fetal aberrations) ^c^Screened population: pregnant women opting for FCT or NIPT.* *Women opting only for the second trimester anomaly scan are not included. ^d^Fetal losses resulting from an invasive test (chorion villus sampling or amniocentesis)*
