# Supplementary material for: Clinical and economic impact of genome-wide non-invasive prenatal testing (NIPT) as a first-tier screening method compared to targeted NIPT and first-trimester combined testing: A modeling study
Source: PLoS Med. 2025 Nov 5;22(11):e1004790. doi: 10.1371/journal.pmed.1004790 (PMC12611151; doi:10.1371/journal.pmed.1004790)
Supplement: S9 Table — (DOCX) [file pmed.1004790.s009.docx]

**S9 Table.** Main outcomes of scenario analysis 3, assuming 100% uptake for all screening options, without a choice in the GW-NIPT strategy (only GW-NIPT is available)

|  | Screening strategy | | | |
| --- | --- | --- | --- | --- |
|  | Second trimester anomaly scan | FCT &  second trimester anomaly scan | Targeted NIPT & second trimester anomaly scan | GW-NIPT & second trimester anomaly scan |
| Fetal T21 diagnosed | 167 | 456 | 533 | 533 |
| Fetal T18 diagnosed | 96 | 135 | 144 | 144 |
| Fetal T13 diagnosed | 38 | 51 | 56 | 56 |
| Other fetal aberrations diagnosed | 46 | 51 | 46 | 139 |
| Total fetal common trisomies diagnosed^a^ | 301 | 642 | 733 | 733 |
| Total fetal diagnosed cases^b^ | 347 | 693 | 779 | 872 |
| Screened population^c^ | 0 | 174,247 | 173,882 | 173,882 |
| Invasive tests | 3,008 | 11,636 | 3,863 | 4,256 |
| Euploid fetal losses^d^ | 3 | 13 | 4 | 4 |
| Invasive tests per fetal case diagnosed | 8.7 | 13.4 | 5.0 | 6.1 |
| Total costs screening program (€) | 58,768,317 | 127,790,660 | 123,881,598 | 127,401,974 |
| Cost per screened individual (€) | - | 733 | 712 | 733 |
| Cost per fetal diagnosed case (€) | 169,361 | 184,402 | 159,026 | 146,271 |
| Incremental cost per additional fetal diagnosed case (ref strategy: scan) (€) |  | 199,487 | 150,725 | 130,731 |
| Incremental cost per additional fetal diagnosed case (ref strategy: FCT) (€) |  |  | -45,454 | -2,171 |
| Incremental cost per additional fetal diagnosed case (ref strategy: targeted NIPT) (€) |  |  |  | 37,854 |

*Abbreviations: FCT. first-trimester combined test; GW. genome-wide; NIPT. non-invasive prenatal testing; T. trisomy.
^a^Sum of all diagnosed fetal T21. T18. and T13
^b^Sum of all diagnosed fetal aberrations (T21. T18. T13. and the other fetal aberrations) ^c^Screened population: pregnant women opting for FCT or NIPT. Women opting only for the second trimester anomaly scan are not included. ^d^Fetal losses resulting from an invasive test (chorion villus sampling or amniocentesis)*
