# Supplementary material for: Clinical and economic impact of genome-wide non-invasive prenatal testing (NIPT) as a first-tier screening method compared to targeted NIPT and first-trimester combined testing: A modeling study
Source: PLoS Med. 2025 Nov 5;22(11):e1004790. doi: 10.1371/journal.pmed.1004790 (PMC12611151; doi:10.1371/journal.pmed.1004790)
Supplement: S10 Table — (DOCX) [file pmed.1004790.s010.docx]

**S10 Table.** Main outcomes of scenario analysis 4, presuming only women of 36 years old or older where invited to genetic screening and assuming 100% of this population opted for FCT or NIPT screening

|  | Screening strategy | | | |
| --- | --- | --- | --- | --- |
|  | Second trimester anomaly scan | FCT &  second trimester anomaly scan | Targeted NIPT & second trimester anomaly scan | GW-NIPT & second trimester anomaly scan |
| Fetal T21 diagnosed | 167 | 344 | 390 | 390 |
| Fetal T18 diagnosed | 96 | 121 | 127 | 127 |
| Fetal T13 diagnosed | 38 | 44 | 46 | 46 |
| Other fetal aberrations diagnosed | 46 | 47 | 46 | 60 |
| Total fetal common trisomies diagnosed^a^ | 301 | 509 | 563 | 563 |
| Total fetal diagnosed cases^b^ | 347 | 556 | 609 | 623 |
| Screened population^c^ | 0 | 33644 | 33573 | 33573 |
| Invasive tests | 3,008 | 4800 | 3352 | 3410 |
| Euploid fetal losses^d^ | 3 | 5 | 3 | 3 |
| Invasive tests per fetal case diagnosed | 8.7 | 8.6 | 5.5 | 5.5 |
| Total costs screening program (€) | 58,768,317 | 72,555,796 | 71,988,539 | 72,653,511 |
| Cost per screened individual (€) | - | 2,157 | 2,144 | 2,164 |
| Cost per fetal diagnosed case (€) | 169,361 | 130,196 | 118,014 | 116,431 |
| Incremental cost per additional fetal diagnosed case (ref strategy: scan) (€) |  | 65,969 | 50,459 | 50,309 |
| Incremental cost per additional fetal diagnosed case (ref strategy: FCT) (€) |  |  | -10,703 | -1,458 |
| Incremental cost per additional fetal diagnosed case (ref strategy: targeted NIPT) (€) |  |  |  | 47,498 |

*Abbreviations: FCT. first-trimester combined test; GW. genome-wide; NIPT. non-invasive prenatal testing; T. trisomy.
^a^Sum of all diagnosed fetal T21. T18. and T13
^b^Sum of all diagnosed fetal aberrations (T21. T18. T13. and the other fetal aberrations) ^c^Screened population: pregnant women opting for FCT or NIPT. Women opting only for the second trimester anomaly scan are not included. ^d^Fetal losses resulting from an invasive test (chorion villus sampling or amniocentesis)*
