# Supplementary figures and images for: Clinical and economic impact of genome-wide non-invasive prenatal testing (NIPT) as a first-tier screening method compared to targeted NIPT and first-trimester combined testing: A modeling study
Source: PLoS Med. 2025 Nov 5;22(11):e1004790. doi: 10.1371/journal.pmed.1004790 (PMC12611151; doi:10.1371/journal.pmed.1004790)

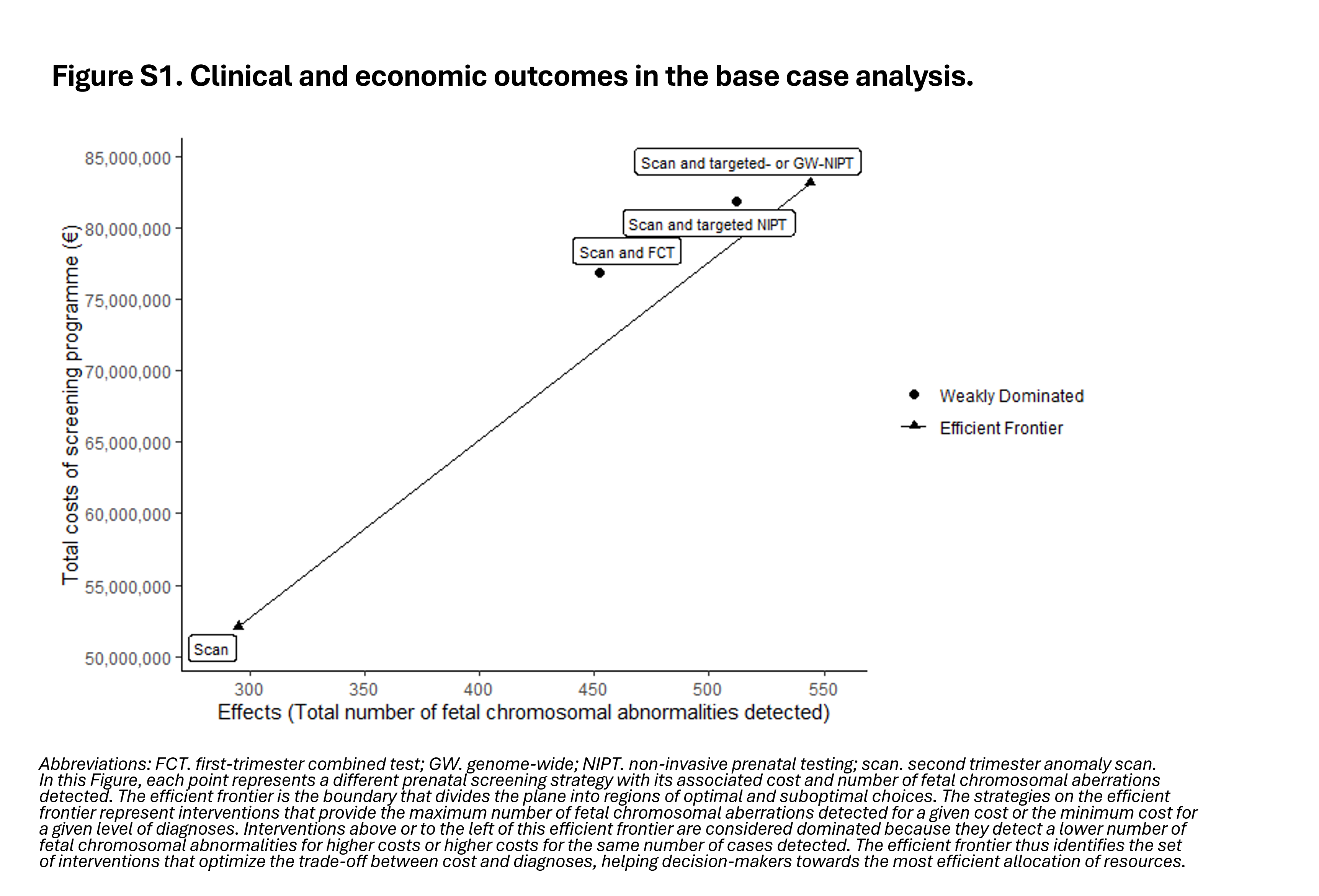

Supplement: S1 Fig — Abbreviations: FCT, first-trimester combined test; GW, genome-wide; NIPT, non-invasive prenatal testing; scan, second-trimester anomaly scan. In this Figure, each point represents a different prenatal screening strategy with its associated cost and number of fetal chromosomal aberrations detected. The efficient frontier is the boundary that divides the plane into regions of optimal and suboptimal choices. The strategies on the efficient frontier represent interventions that provide the maximum number of fetal chromosomal aberrations detected for a given cost or the minimum cost for a given level of diagnoses. Interventions above or to the left of this efficient frontier are considered dominated because they detect a lower number of fetal chromosomal abnormalities for higher costs or higher costs for the same number of cases detected. The efficient frontier thus identifies the set of interventions that optimize the trade-off between cost and diagnoses, helping decision-makers towards the most efficient allocation of resources. (TIF) [file pmed.1004790.s011.tif]

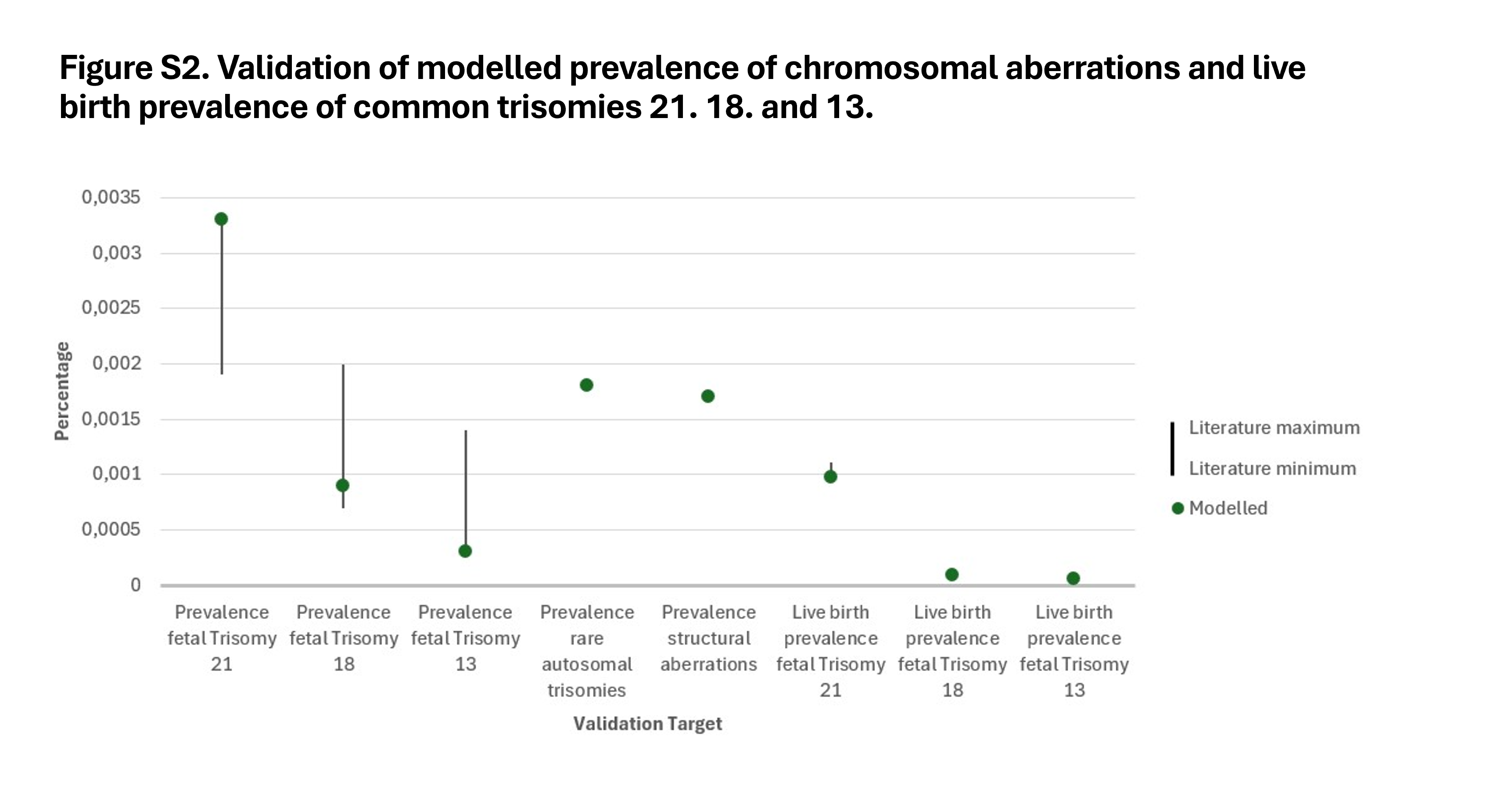

Supplement: S2 Fig — The dot shows the modeled value. The line indicates the range from the minimum to maximum parameter values based on the literature. The prevalence of autosomal trisomies and structural aberrations is based on TRIDENT-2 data and was assumed in the model; therefore, the min–max line coincides with the modeled parameter value. (TIF) [file pmed.1004790.s012.tif]

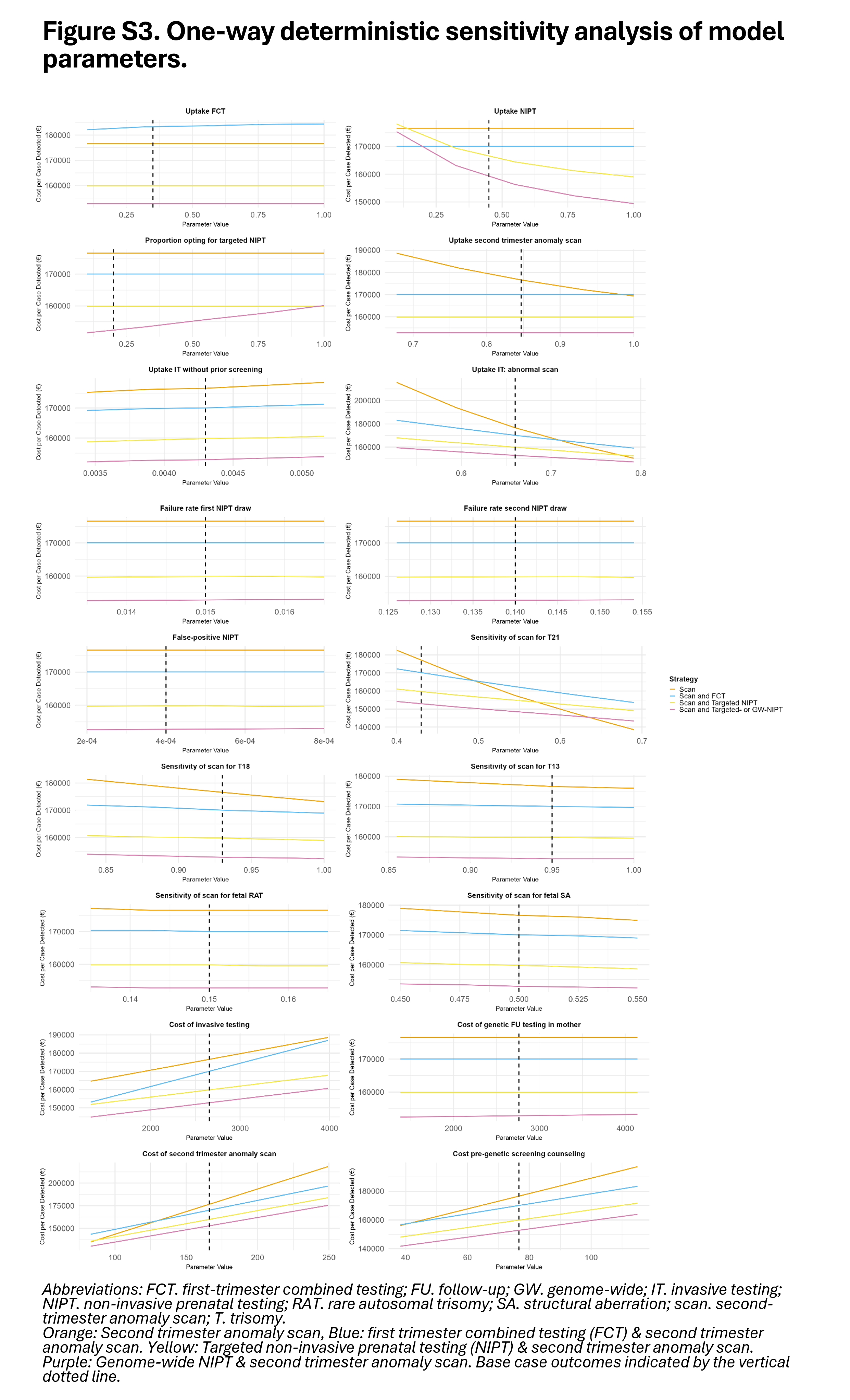

Supplement: S3 Fig — Abbreviations: FCT, first-trimester combined testing; FU, follow-up; GW, genome-wide; IT, invasive testing; NIPT, non-invasive prenatal testing; RAT, rare autosomal trisomy; SA, structural aberration; scan, second-trimester anomaly scan; T, trisomy. Orange: Second-trimester anomaly scan, Blue: first-trimester combined testing (FCT) and second-trimester anomaly scan, Yellow: Targeted non-invasive prenatal testing (NIPT) and second-trimester anomaly scan, Purple: Genome-wide NIPT and second-trimester anomaly scan. Base case outcomes indicated by the vertical dotted line. (TIF) [file pmed.1004790.s013.tif]

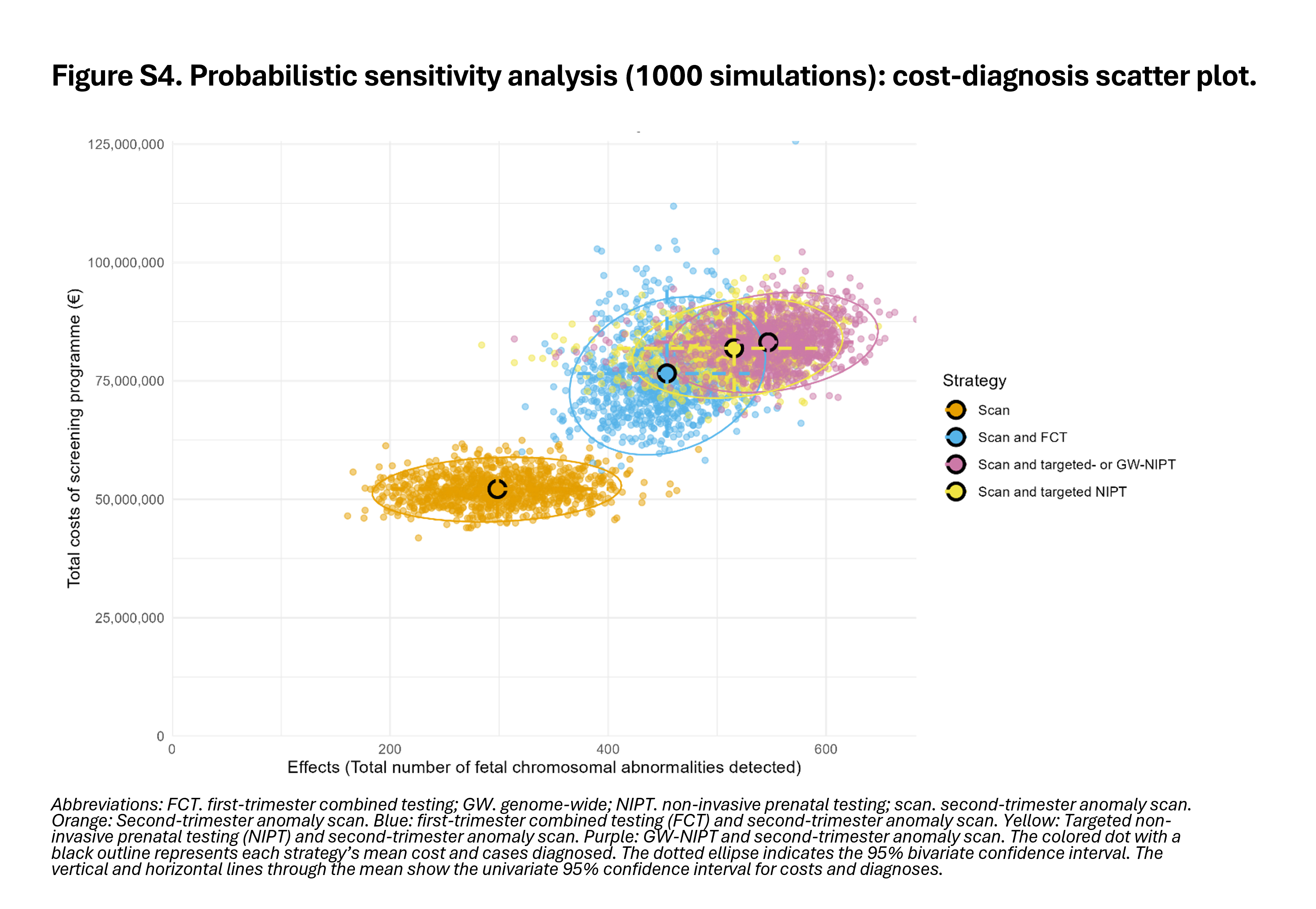

Supplement: S4 Fig — Abbreviations: FCT, first-trimester combined testing; GW, genome-wide; NIPT, non-invasive prenatal testing; scan, second-trimester anomaly scan. Orange: Second-trimester anomaly scan, Blue: first-trimester combined testing (FCT) and second-trimester anomaly scan, Yellow: Targeted non-invasive prenatal testing (NIPT) and second-trimester anomaly scan, Purple: GW-NIPT and second-trimester anomaly scan. The colored dot with a black outline represents each strategy’s mean cost and cases diagnosed. The dotted ellipse indicates the 95% bivariate confidence interval. The vertical and horizontal lines through the mean show the univariate 95% confidence interval for costs and diagnoses. (TIF) [file pmed.1004790.s014.tif]
