# Supplementary material for: Clinical and economic impact of genome-wide non-invasive prenatal testing (NIPT) as a first-tier screening method compared to targeted NIPT and first-trimester combined testing: A modeling study
Source: PLoS Med. 2025 Nov 5;22(11):e1004790. doi: 10.1371/journal.pmed.1004790 (PMC12611151; doi:10.1371/journal.pmed.1004790)
Supplement: S1 Checklist — The checklist is Open Access distributed in accordance with the terms of the Creative Commons Attribution (CC BY 4.0) license. (DOCX) [file pmed.1004790.s015.docx]

**CHEERS 2022 Checklist**

|  | **Item** | **Guidance for Reporting** | **Reported in section** |
| --- | --- | --- | --- |
| **TITLE** | | | |
| Title | 1 | Identify the study as an economic evaluation and specify the interventions being compared. | p1, lines 1-4 |
| **ABSTRACT** | | | |
| Abstract | 2 | Provide a structured summary that highlights context, key methods, results and alternative analyses. | P3-4, lines 46-75 |
| **INTRODUCTION** | | | |
| Background and objectives | 3 | Give the context for the study, the study question and its practical relevance for decision making in policy or practice. | P8-9, lines 139-178 |
| **METHODS** | | | |
| Health economic analysis plan | 4 | Indicate whether a health economic analysis plan was developed and where available. | No structured plan was established; however, all the items listed below were addressed, and the Decision Analysis in R for Technologies in Health (DARTH) framework was utilized for the analysis. |
| Study population | 5 | Describe characteristics of the study population (such as age range, demographics, socioeconomic, or clinical characteristics). | p12 (‘*Population’* & Table 1) & Maternal age distribution and risk for chromosomal abnormalities in Table S1. |
| Setting and location | 6 | Provide relevant contextual information that may influence findings. | p9, Introduction, lines 172-178  p10, Materials and Methods ‘Screening strategies’, and p12, ‘Population’ |
| Comparators | 7 | Describe the interventions or strategies being compared and why chosen. | p10. ‘screening strategies’ |
| Perspective | 8 | State the perspective(s) adopted by the study and why chosen. | p16. ‘costs’ |
| Time horizon | 9 | State the time horizon for the study and why appropriate. | p16. One year, duration of pregnancy |
| Discount rate | 10 | Report the discount rate(s) and reason chosen. | p16. No discount rate was applied, as the analysis focused on a one-year horizon (the duration of pregnancy). |
| Selection of outcomes | 11 | Describe what outcomes were used as the measure(s) of benefit(s) and harm(s). | p16. ‘outcomes’ |
| Measurement of outcome | 12 | Describe how outcomes used to capture benefit(s) and harm(s) were measured. | p16. ‘outcomes’ |
| Validation of outcomes | 13 | Describe the population and methods used to measure and value outcomes. | p16. ‘outcomes’, Table S5 and Figure S2 |
| Measurement and valuation of resources and costs | 14 | Describe how costs were valued. | p16. ‘costs’ |
| Currency, price date, and conversion | 15 | Report the dates of the estimated resource quantities and unit costs, plus the currency and year of conversion. | p16. ‘costs’ |
| Rationale and description of model | 16 | If modelling is used, describe in detail and why used. Report if the model is publicly available and where it can be accessed. | p10. ‘the model’ &  p12. ‘Analysis’ &  p28. ‘Data & code availability’ |
| Analytics and assumptions | 17 | Describe any methods for analysing or statistically transforming data, any extrapolation methods, and approaches for validating any model used. | p12. ‘population’ &  p16. ‘model validation’ |
| Characterizing heterogeneity | 18 | Describe any methods used for estimating how the results of the study vary for sub-groups | p17. ‘scenario analysis’ results are presented for the population aged 36 and older. Other outcomes focused on the overall population. |
| Characterizing distributional effects | 19 | Describe how impacts are distributed across different individuals or adjustments made to reflect priority populations. | p17. ‘scenario analysis’ results are presented for the population aged 36 and older. Other outcomes focused on the overall population. |
| Characterizing uncertainty | 20 | Describe methods to characterize any sources of uncertainty in the analysis. | p17. ‘robustness of outcomes’ |
| Approach to engagement with patients and others affected by the study | 21 | Describe any approaches to engage patients or service recipients, the general public, communities, or stakeholders (e.g., clinicians or payers) in the design of the study. | p10. ‘the model’ > The modelled pathways were developed with input from relevant specialists, including clinical geneticists, lab specialists, and gynecologists. |
| **RESULTS** | | | |
| Study parameters | 22 | Report all analytic inputs (e.g., values, ranges, references) including uncertainty or distributional assumptions. | Table 1 |
| Summary of main results | 23 | Report the mean values for the main categories of costs and outcomes of interest and summarise them in the most appropriate overall measure. | Table 3 |
| Effect of uncertainty | 24 | Describe how uncertainty about analytic judgments, inputs, or projections affect findings. Report the effect of choice of discount rate and time horizon, if applicable. | p20-22. ‘robustness of outcomes’ |
| Effect of engagement with patients and others affected by the study | 25 | Report on any difference patient/service recipient, general public, community, or stakeholder involvement made to the approach or findings of the study. | No direct results were obtained, but the modelled pathways were developed and validated through discussions with specialists. |
| **DISCUSSION** | | | |
| Study findings, limitations, generalizability and current knowledge | 26 | Report key findings, limitations, ethical or equity considerations not captured, and how these could impact patients, policy, or practice. | Key findings: Lines 475-482, Limitations and impact: Lines 524-549 |
| **OTHER RELEVANT INFORMATION** | | | |
| Source of funding | 27 | Describe how the study was funded and any role of the funder in the identification, design, conduct, and reporting of the analysis. | No funding was received for this study |
| Conflicts of interest | 28 | Report authors conflicts of interest according to journal or International Committee of Medical Journal Editors requirements. | p28: Disclosure: The authors declare no conflict of interest. |

Husereau D, Drummond M, Augustovski F, de Bekker-Grob E, Briggs AH, Carswell C, Caulley L, Chaiyakunapruk N, Greenberg D, Loder E, Mauskopf J, Mullins CD, Petrou S, Pwu RF, Staniszewska S; CHEERS 2022 ISPOR Good Research Practices Task Force. Consolidated Health Economic Evaluation Reporting Standards 2022 (CHEERS 2022) Statement: Updated Reporting Guidance for Health Economic Evaluations. BMJ. 2022;376:e067975. The checklist is Open Access distributed in accordance with the terms of the Creative Commons Attribution (CC BY 4.0) license, which permits others to distribute, remix, adapt and build upon this work, for commercial use, provided the original work is properly cited. See: http://creativecommons.org/licenses/by/4.0/.
